# Tissue Pharmacokinetic Properties and Bystander Potential of Hypoxia-Activated Prodrug CP-506 by Agent-Based Modelling

**DOI:** 10.3389/fphar.2022.803602

**Published:** 2022-02-08

**Authors:** Victoria Jackson-Patel, Emily Liu, Matthew R. Bull, Amir Ashoorzadeh, Gib Bogle, Anna Wolfram, Kevin O. Hicks, Jeff B. Smaill, Adam V. Patterson

**Affiliations:** ^1^ Auckland Cancer Society Research Centre, Faculty of Medical and Health Sciences, University of Auckland, Auckland, New Zealand; ^2^ Maurice Wilkins Centre for Molecular Biodiscovery, University of Auckland, Auckland, New Zealand; ^3^ Auckland Bioengineering Institute, University of Auckland, Auckland, New Zealand

**Keywords:** CP-506, SN36506, PR-104 (PubChem CID: 11455973), PR-104A (PubChem CID: 9848786), bystander effect, hypoxia-activated prodrug, agent-based modelling (ABM), multicellular spheroids

## Abstract

Hypoxia-activated prodrugs are bioactivated in oxygen-deficient tumour regions and represent a novel strategy to exploit this pharmacological sanctuary for therapeutic gain. The approach relies on the selective metabolism of the prodrug under pathological hypoxia to generate active metabolites with the potential to diffuse throughout the tumour microenvironment and potentiate cell killing by means of a “bystander effect”. In the present study, we investigate the pharmacological properties of the nitrogen mustard prodrug CP-506 in tumour tissues using *in silico* spatially-resolved pharmacokinetic/pharmacodynamic (SR-PK/PD) modelling. The approach employs a number of experimental model systems to define parameters for the cellular uptake, metabolism and diffusion of both the prodrug and its metabolites. The model predicts rapid uptake of CP-506 to high intracellular concentrations with its long plasma half-life driving tissue diffusion to a penetration depth of 190 µm, deep within hypoxic activating regions. While bioreductive metabolism is restricted to regions of severe pathological hypoxia (<1 µM O_2_), its active metabolites show substantial bystander potential with release from the cell of origin into the extracellular space. Model predictions of bystander efficiency were validated using spheroid co-cultures, where the clonogenic killing of metabolically defective “target” cells increased with the proportion of metabolically competent “activator” cells. Our simulations predict a striking bystander efficiency at tissue-like densities with the *bis*-chloro-mustard amine metabolite (CP-506M-Cl_2_) identified as a major diffusible metabolite. Overall, this study shows that CP-506 has favourable pharmacological properties in tumour tissue and supports its ongoing development for use in the treatment of patients with advanced solid malignancies.

## Introduction

Heterogeneous tumour oxygenation has long been recognized as a major impediment to the development of effective cancer therapies (Hockel et al., 1996; Nordsmark et al., 2005; Wilson and Hay, 2011; Marusyk et al., 2012). Given the prevalence of tumour hypoxia (Fukumura and Jain, 2007; Pries et al., 2009) and its association with treatment failure (Vaupel and Mayer, 2007), the use of hypoxia-activated prodrugs is a noteworthy approach to improve clinical outcomes. Hypoxia-activated prodrugs are designed to preferentially target regions of severe hypoxia, allowing tumour dose intensification by circumventing the normal tissue toxicities (Denny, 2005; Denny, 2010) that traditionally limit the potential for dose-escalation with conventional chemotherapeutic regimens (Rowinsky, 2000; Crawford, 2013).

Essentially, most hypoxia-activated prodrugs are comprised of a bioreductive switch or trigger unit that deactivates or masks the cytotoxic effector under aerobic conditions. Computational studies have elegantly shown that the efficacy of a hypoxia-activated prodrug is a delicate balance between its cellular affinity (reversible binding or sequestration), metabolic stability (prodrug activation rate and O_2_-dependence), potency and diffusion capabilities of the released effector (Hicks et al., 1998; Hicks et al., 2003; Foehrenbacher et al., 2013a; Foehrenbacher et al., 2013b; Hong et al., 2018a; Hong et al., 2019). In which, the prodrug must penetrate relatively long distances through the extravascular compartment in order to reach the cells that are sufficiently hypoxic for its metabolic activation and subsequent cytotoxicity. The localization of the hypoxic target cells and the relative degree of effector redistribution (known as the bystander effect) determine the resultant cell killing. The limited success of earlier clinical candidates reflects, in part, the complexity of this design criteria (Marcu and Olver, 2006; Spiegelberg et al., 2019; Li et al., 2021).

A noteworthy example is the nitroaromatic compound PR-104A. Experimental studies showed that PR-104A is readily metabolised by endogenous one-electron reductases (e.g. cytochrome P450 oxidoreductase (POR) and other diflavin oxidoreductases) into a radical species that spontaneously converts under hypoxic conditions into DNA cross-linking metabolites (notably hydroxylamine PR-104H and amine PR-104M) (Guise et al., 2007; Patterson et al., 2007; Singleton et al., 2009; Guise et al., 2012). Computational studies indicated favourable pharmacokinetic properties for both single agent and combinatorial activity owing to its strict oxygen dependence (the half-maximal activation occurs at *K*O_2_ = 0.126 µM O_2_) and bystander efficiency in experimental models (Hicks et al., 2007; Foehrenbacher et al., 2013a; Foehrenbacher et al., 2013b). A sizeable bystander effect was shown using various multicellular layer (MCL) (Foehrenbacher et al., 2013a), spheroid co-culture (Hong et al., 2018b) and tumour xenograft models (Patterson et al., 2007; Foehrenbacher et al., 2013a). Combinatorial activity was noted with radiation (Hicks et al., 2007) as well as various systemic agents (Patterson et al., 2007; Abbattista et al., 2015).

Despite its promising preclinical activity, PR-104A, when administered to patients as its phosphate pre-prodrug form PR-104, failed to demonstrate significant clinical efficacy due to its myelotoxicity profile. The principal toxicities of neutropenia and thrombocytopenia aligned with those of conventional therapies resulting in additive toxicity rather than efficacy upon co-administration (Jameson et al., 2010; Abou-Alfa et al., 2011; Mckeage et al., 2011; Mckeage et al., 2012). A retrospective study identified an alternative route of PR-104A activation involving two-electron metabolism by aldo-keto reductase 1C3 (AKR1C3) that bypasses the oxygen-dependent step in the activation schematic (Guise et al., 2010). The off-target activation of PR-104A was specific to the human AKR1C3 orthologue explaining the clear oversight during its preclinical development (Guise et al., 2010; Van Der Wiel et al., 2021).

CP-506 is a second-generation PR-104A analogue that is rationally designed to be resistant to AKR1C3 activation in human tissues (Van Der Wiel et al., 2021). The mechanism of activation involves an initial one-electron reduction by endogenous oxidoreductases to a nitro radical anion intermediate that acts as a direct oxygen sensor, as previously described for PR-104A (Patterson et al., 2007). Experimental studies have shown that this reaction is catalysed efficiently by cytochrome P450 oxidoreductase (POR), although a number of other flavoenzymes can also contribute (Van Der Wiel et al., 2021). Further reduction leads to the formation of various DNA cross-linking metabolites selectively under hypoxic conditions. While CP-506 shows similar potency to PR-104A in anti-proliferative assays, the oxygen-dependence of prodrug activation and cytotoxicity is more favourable with complete inhibition above 1 µM O_2_ and through the physiological O_2_ concentration range (Van Der Wiel et al., 2021).

In the present study, we investigate the extravascular transport properties and bystander efficiency of CP-506 in tumour tissues by coupling various experimental and computational modelling approaches. The experimental framework involves a series of novel *in vitro* systems to measure reaction-diffusion in cells and 3D tumour models. Then, a well-validated in silico spatially-resolved pharmacokinetic/pharmacodynamic (SR-PK/PD) modelling approach (Hicks et al., 1997; Hicks et al., 2006; Hicks et al., 2010; Foehrenbacher et al., 2013b) was applied to estimate parameters for the cellular uptake, metabolism and diffusion of CP-506 and its active metabolites in the tumour tissue (Foehrenbacher et al., 2013a; Hong et al., 2018b). A cellular PK model was first developed by quantifying the extracellular (C_e_) to intracellular (C_i_) partitioning ratio for CP-506 and its formed metabolites in monolayer cultures treated under normoxic (21% O_2_) and anoxic conditions (<1 ppm O_2_). The derived reaction and diffusion terms were then scaled to tissue-like densities based on the transport of the prodrug and its effectors across multicellular layer (MCL) cultures maintained under supraoxic (95% O_2_) and hypoxic conditions (<1 ppm O_2_). Next, the parameter estimates were validated by comparing theoretical predictions to experimental determinations of clonogenic cell killing in spheroid co-cultures by agent-based modelling (ABM) (Hong et al., 2018b). Differences in fluorescent protein expression (mRuby vs. EGFP), antibiotic resistance genes (puromycin vs. geneticin) and POR expression (high vs. null) were used to delineate between the subpopulations of metabolically competent “activator” and metabolically defective “target” cells to interpret experimental findings. We conclude by simulating the spatial gradients of CP-506 and its active metabolites in a transverse section of a representative spheroid. Our findings illustrate the superior tissue pharmacokinetic properties of CP-506 relative to PR-104A and support its future clinical evaluation in patients with advanced solid malignancies.

## Materials and Methods

### Compounds

CP-506 (2-[(2-bromoethyl)-5-[(4-ethyl-1-piperazinyl)carbonyl]-2-(methylsulfonyl)-4-nitro anilino]ethyl methanesulfonate), CP-506H (2-((2-bromoethyl)(5-(4-ethylpiperazine-1-carbonyl)-4-(hydroxyamino)-2-(methylsulfonyl)phenyl)amino)ethyl methanesulfonate) and CP-506M (2-((4-amino-5-(4-ethylpiperazine-1-carbonyl)-2-(methylsulfonyl)phenyl)(2-bromoethyl)amino)ethyl methanesulfonate) and their respective deuterated (D8) standards (prepared from D8-1-ethylpiperazine) were manufactured by Mercachem (Nijmegen, the Netherlands) employing a synthetic route developed at the University of Auckland as previously described (Van Der Wiel et al., 2021). The downstream metabolites CP-506H-(OH)_2_ (5-(bis(2-hydroxyethyl)amino)-2-(hydroxyamino)-4-(methylsulfonyl)phenyl)(4-ethylpiperazin-1-yl)methanone), CP-506M-(OH)_2_ (2-amino-5-(bis(2-hydroxyethyl)amino)-4-(methylsulfonyl)phenyl)(4-ethylpiperazin-1-yl)methanone), CP-506H-Cl_2_ (5-(bis(2-chloroethyl)amino)-2-(hydroxyamino)-4-(methylsulfonyl)phenyl)(4-ethylpiperazin-1-yl)methanone), CP-506M-Cl_2_ (2-amino-5-(bis(2-chloroethyl)amino)-4-(methylsulfonyl)phenyl)(4-ethylpiperazin-1-yl)methanone) and their respective deuterated (D8) standards (prepared from D8-diethanolamine) were synthesized at the University of Auckland as described in the supplementary methods. Stock solutions were prepared in DMSO (CP-506, CP-506H and CP-506M) or acetonitrile (CP-506H-Cl_2_, CP-506H-(OH)_2_, CP-506M-Cl_2_, CP-506M-(OH)_2_) to concentrations of up to 100 mM and stored at −80°C until use. Working solutions were made by dilution into culture medium immediately before drug addition on the day of the experiment.

### Cell Culture

The HCT-116 cell line was purchased from the American Type Culture Collection (Manassas, VA). A POR-overexpressing clone (POR-R) was generated by transfecting the parental cell line with a pBRP expression vector encoding an N-terminal truncated soluble version of the human POR gene (Foehrenbacher et al., 2013a; Guise et al., 2020). Ectopic expression is driven by the human cytomegalovirus (CMV) major immediate-early promoter. Downstream of POR is a human elongation factor-1 alpha (EF-1 alpha) promoter, which drives expression of mRuby and the puromycin resistance gene *pac* (puromycin N-acetyltransferase). A POR-null clone (PORG-ko) was generated as previously described (Su et al., 2013). POR-null clones were then transfected with a pEGFP-N1 plasmid encoding enhanced green fluorescent protein (EGFP) and geneticin resistance to permit identification upon co-culture (Hong et al., 2018b). Cell lines were maintained by weekly subculture in T-flasks containing alpha minimal essential medium (αMEM) with 5% foetal bovine serum (FBS) for a maximum of 24 passages or 90 days (whichever came first). The culture medium was further supplemented with 1 mg/ml geneticin or 2 µM puromycin for the routine maintenance of the POR-null and POR-overexpressing clones respectively. All cultures were re-established from STR-authenticated, mycoplasma negative frozen stocks.

### Anti-Proliferative IC_50_ Assays

Cells (700–1,000 per well) were seeded with 100 µl culture medium into 96-well plates and left to attach for 2 h in a humidified 37°C incubator. Compounds were prepared in culture medium and serially diluted (1:3) in the well volume. Plates were returned to a humidified 37°C incubator for the duration of the drug exposure (4 h). Aerobic experiments were performed under standard tissue culture conditions (21% O_2_), and the anoxic experiments were performed in parallel in a 5% H_2_/Pd catalyst anaerobic chamber (Shellab Bactron, Sheldon manufacturing Inc., Cornelius, OR) using pre-equilibrated media and plasticware. All anoxia experiments were conducted using a catalyst-scrubbed anaerobic chamber, unless otherwise specified, with gas oxygen measurements of <1 ppm O_2_. Cells were then washed three times and left to grow for 96 h before cell density determination using a sulforhodamine B (SRB) assay (Vichai and Kirtikara, 2006). To this end, the cells were fixed by layering 50 µl of trichloroacetic acid (40% w/v in MilliQ water) on top of the culture media (150 µl) in each well for 1 h at 4°C. Plates were rinsed under running tap water and stained with 50 µl of SRB (0.4% w/v in MilliQ water containing 1% v/v acetic acid) for 30 min at room temperature. Plates were rapidly destained by rinsing three times in tap water containing 1% (v/v) acetic acid. The stain was solubilized by adding 100 µl of 10 mM unbuffered Tris base to each well with gentle rocking for 1 h. Plates were read on a Biotek ELx808 Absorbance Microplate Reader at 490 nm using KC4 software. The IC_50_ value (concentration required to reduce staining to 50% of the untreated control on the same plate) for each compound was determined by interpolation using a 4-parameter logistic regression model.

### Monolayer Clonogenic Assays

Cells (4.8 x 10^5^ cells) were seeded with 2 ml culture medium into 6-well plates and left to attach for 2 h in a humidified 37°C incubator. Compounds were then prepared in culture medium and added in a 500 µl volume to each well. Plates were returned to a humidified 37°C incubator for the duration of the drug exposure (4 h). Aerobic and anoxic experiments were performed as described above. Plates were then removed from the anaerobic chamber and processed alongside the aerobic cultures. Cells were detached from monolayer by trypsinisation and plated at serial dilutions in 6-well plates. Cells were left to grow in a standard 37°C incubator for 10 days until the controls formed discrete colonies comprised of at least 50 cells. Colonies were visualized by staining with methylene blue (2 g/L in 50% aqueous ethanol) for 30 min. The number of colonies with at least 50 cells was manually counted to determine the plating efficiency. The surviving fraction was determined as the ratio of plating efficiency of treated cells to that of the controls.

### Cellular Metabolism Assay

Aerobic and anoxic conditions were achieved as described above. Cells (5 x 10^5^ per well) were seeded with 350 µl medium in duplicate into 24-well plates and left to attach for 2 h in a humidified 37°C incubator. A working solution of CP-506 was prepared in culture medium to achieve a 100 µM concentration in each well after the addition of a 500 µl volume. Plates were exposed to drug for 1 h in a humidified 37°C incubator. Metabolism was then halted by the addition of two-volumes of ice-cold acetonitrile containing 2 µM of internal standard for each analyte. Samples were stored at −80°C until LC-MS/MS analysis.

### Media Stability Studies

The stability of each compound was determined in stirred medium (without serum) maintained under aerobic conditions by the constant supply of a humidified gas mixture comprised of 21% O_2_, 5% CO_2_ with balance N_2_. Vials were spiked with 100 µM of the relevant compound, and samples were collected using a positive displacement pipettor for up to 3 h thereafter. The collected samples were then treated with two-volumes of ice-cold acetonitrile containing 2 µM of the relevant deuterated (D8) internal standard and stored at −80°C until LC-MS/MS analysis.

### Cellular Uptake Studies

The intracellular/extracellular partitioning ratio for CP-506 and its major metabolites was determined in monolayer cultures of POR-overexpressing HCT-116 cells, as described previously (Hong et al., 2018b). Briefly, cells (5 x 10^5^ per well) were seeded with 350 µl medium into 24-well plates and left to attach for 2 h in a humidified 37°C incubator. Stock solutions of CP-506 were diluted and added with 50 µl medium to the well volume to achieve a final concentration of 100 µM. Samples were harvested at various time points for up to 3 h thereafter. Aerobic experiments were performed under standard tissue culture conditions (21% O_2_), and the anoxic experiments in parallel in a H_2_/Pd-catalyst-scrubbed anaerobic chamber as described. The well volume was spiked with [^3^H]-mannitol immediately before sample harvest and treated with two-volumes of ice-cold acetonitrile containing 2 µM of the deuterated (D8) internal standards for each CP-506, CP-506H and CP-506M. The cell monolayer was then treated with 100 µl of this crashing solution. Samples were stored at −80°C until LC-MS/MS analysis. The cellular exclusion of [^3^H]-mannitol was used to account for the contribution of the extracellular medium toward determinations of the intracellular concentration, as described previously (Foehrenbacher et al., 2013a).

### Multicellular Layer Flux Studies

The extravascular transport properties of CP-506 and its formed metabolites were investigated using a custom-designed diffusion apparatus, as described previously (Hicks et al., 2006). Briefly, MCL cultures were established by seeding 1 x 10^6^ POR-R cells onto collagen-coated Millicell-CM inserts for 3 days. Established MCLs were placed at the interface of the two media-filled compartments of diffusion chambers. Diffusion chambers were placed in a 37°C water bath and gassed with a supraoxic (95% O_2_, 5% CO_2_) or anoxic (5% CO_2_, bal N_2_) mixture for at least 1 h prior to drug addition. Flux was initiated by the addition of internal standard ([^14^C]-urea) and 20 µM CP-506 to the donor compartment. Samples (100 µl) were collected from both the donor and receiver compartments for up to 5 h thereafter. At each time point, a 25 µl aliquot was mixed with emulsifier-safe water-accepting scintillant and assayed for radioactivity using a Perkin Elmer Tricard 2910 TR liquid scintillation analyser. The remaining sample was treated with two volumes of ice-cold acetonitrile (containing 2 µM of the relevant deuterated internal standards) and stored at −80°C until LC-MS/MS analysis. Similar experiments were performed using collagen-coated inserts without MCLs to deduce the chemical stability and diffusion coefficient of CP-506 across the bare support membrane.

### Spheroid Growth and Survival Assays

Spheroid cultures were established by seeding 3 x 10^3^ cells with 100 µl medium into ultra-low attachment, round bottom 96-well plates (Corning Inc, Corning, NY). Plates were briefly centrifuged (200 g, 5 min) to facilitate cell aggregation and grown in a standard incubator (37°C, 21% O_2_, 5% CO_2_) for 4 days before experiments. The culture medium was then aspirated, and the established spheroids taken into a H_2_/Pd-catalyst anaerobic chamber. Spheroids were washed three times with anoxia pre-equilibrated medium and left to settle for 1 h in a humidified 37°C incubator. Spheroids were removed from the anaerobic chamber after a 4 h drug exposure and washed three times with fresh culture medium. The growth of representative spheroids was monitored for 10 days using a published method of image analysis for volume determination (Mao et al., 2018). The remainder were enzymatically dissociated and plated at serial dilutions in 6-well plates containing 2 µM puromycin or 1 mg/ml geneticin for positive selection of POR-R and PORko-G cells, respectively. Colonies were visualized after 10 days of drug-free proliferation by staining with methylene blue (2 g/L in 50% aqueous ethanol) for 30 min. The number of colonies with at least 50 cells was manually counted to determine the plating efficiency. The surviving fraction was determined as the ratio of the plating efficiency of treated cells to that of the controls.

### Flow Cytometric Detection of EF5 Binding in Multicellular Spheroids

Spheroid cultures were established by seeding 3 x 10^3^ cells into ultra-low attachment, round bottom 96-well plates, as described above. The culture medium was then aspirated, and the established spheroids taken into the H_2_/Pd-catalyst anaerobic chamber. Spheroids were washed three times with anoxia pre-equilibrated medium and left to settle for 1 h in a humidified 37°C incubator. Spheroids were then treated with 100 µM EF5 for 4 h at 37°C. Plates were removed from the anaerobic chamber and washed three times with fresh culture medium. Spheroids were then dissociated by trypsinisation, washed three times with 0.1 M PBS and fixed in 4% formaldehyde. Samples were blocked with PBS containing 0.3% Tween-20 (PBS-T) and 10% FBS for 1 h at 4°C. EF5 adducts were then stained with 10 μg/ml Alexa Fluor 488-conjugated Elk3-51 antibody (supplied by Prof. Cameron Koch, University of Pennsylvania) diluted in incubation buffer (5% FBS, PBS-T *v/v*) overnight at 4°C. After a further three washes in PBS-T, cells were stored in 500 μl of PBS at 4°C until analysis on a BD Accuri flow cytometer (BD Biosciences, Franklin Lakes, NJ). A primary gate was set based on forward and side scatter to exclude cellular debris, dead cells and doublets. A secondary gate was set based on the selective metabolism and binding of EF5 in monolayer cultures treated under anaerobic conditions (<1 ppm O_2_) compared to standard tissue culture conditions (37°C, 21% O_2_, 5% CO_2_) to define EF5-positive events.

### Flow Cytometric Analysis of Fluorescent Protein Expression

The relative proportion of POR-R (“activator”) and PORko-G (“target”) cells in spheroid co-cultures was quantified by the flow cytometric detection of EGFP expression in paired samples. Established spheroids were enzymatically dissociated and re-suspended in 500 µl of 0.1 M PBS for analysis on a BD Accuri flow cytometer (BD Biosciences, Franklin Lakes, NJ). A primary gate was set based on forward and side scatter to exclude cellular debris, dead cells and doublets. A secondary gate was set based on the basal fluorescence of the parental HCT-116 cell line to define GFP-positive events.

### Western Immunoblotting

Cellular lysates were prepared from log-phase cultures using modified radioimmuno-precipitation assay (RIPA) buffer (50 mM Tris-HCl, 1% NP-40, 0.25% sodium deoxycholate, 150 mM NaCl, 1mM EDTA, 1 mM Na_3_VO_4_, 1 mM NaF, 1:100 protease inhibitor cocktail (Roche, Basel, Switzerland)). Cellular lysates were then diluted in sample buffer (4 x LDS sample buffer containing 5% β-mercaptoethanol) and loaded with equal amounts of protein (30 µg) into BOLT precast gels (Thermofisher Scientific, Carlsbad, CA). Proteins were separated at 100 V for 1 h using BOLT MES SDS running buffer (Thermofisher Scientific, Carlsbad, CA) and transferred at 100 V for 1 h onto polyvinylidene difluoride (PVDF) membranes (pre-soaked in 100% methanol for 5 min) using ice-cold transfer buffer (14.4 g glycine, 3 g Trisma base, 200 ml methanol, 800 ml MilliQ water). Membranes were incubated with blocking solution for 1 h followed by the relevant primary antibody overnight at 4^o^C. Membranes were then washed three times with 0.1 M TBS-T (48 g Tris-HCl, 11.2 g Trisma base, 176 g NaCl, 20 ml Tween-20, 1800 ml MilliQ water (*w/v*)) and incubated with the appropriate HRP-conjugated secondary antibody for 1 h at room temperature. After a further three washes with 0.1 M TBS-T, bands were visualized with Supersignal West Pico Chemiluminescent substrate (Thermofisher Scientific, Carlsbad, CA) using a ChemiDoc MP Imaging System (Biorad Laboratories, Hercules, CA). Band densitometry was performed using Image J software (Schneider et al., 2012). Additional details regarding the antibodies and blocking solutions used in this study can be found in Supplementary Table S1.

### LC-MS/MS Analysis of CP-506 and Metabolites

The LC system for tandem MS consisted of an Agilent 1200 autosampler (4°C) and binary pump, a 3.0 × 50 mm, 1.8-micron Zorbax SB-C18 column (Agilent; PN: 827975-302) and 2.1 × 7 mm guard column (Alltech, IL) maintained at 35°C, at a pressure of <400 bar and flow rate of 0.3 ml/min. The mobile phase comprised a gradient of 0.01% formic acid in 80% acetonitrile - 20% water, v/v (solvent A), and 0.01% formic acid in water (solvent B). Initial mobile phase composition was 20% solvent A and 80% solvent B, increasing to 60% A (0–3 min) then 80% A (3–5.45 min), returning to initial conditions (5.5–6 min). Post-run time was 1 min, and the total run time was 9 min. Mass spectrometric detection was carried out using an Agilent 6410 triple quadrupole mass spectrometer equipped with a multimode ionization (MMI) source. The mass spectrometer was operated in electrospray positive ionization mode using multiple reaction monitoring (MRM), with Q1 & Q3 set to unit resolution (0.7 μm). The electrospray ionization parameters, optimized for the parent molecular ion ([M+H]+) abundance were: drying gas temperature 325°C, vaporiser temperature 150°C, drying gas flow 5 L/min, nebuliser pressure 50 psi, capillary voltage 1.8 kV, Delta EMV 300 V. Ultra-pure nitrogen was used as collision gas. The divert valve feature of the Agilent 6410 triple quadrupole mass spectrometer was utilised to prevent the eluate from entering the ionisation source chamber during the first 0.5 min of each run. For CP-506 samples, positively charged ions representing the [M+H]+ for CP-506 and CP-506-D8 were collisionally dissociated to form specific product ions which were monitored in MS2 (unit resolution). The MRM transition, ionization mode, collision energy, dwell time, fragmentor voltage and retention time for all compounds is provided in Supplementary Table S2. Agilent MassHunter software (v.4.04.00) was used for data acquisition and chromatographic peak integration.

### Cellular Pharmacokinetic Model

An agent-based model (ABM) employing a one-dimensional approximation for monolayers was used to develop a cellular PK model, as described in further detail elsewhere (Hong et al., 2018b; Mao et al., 2018). The mathematical framework assumes that solute concentrations in the medium are dependent only on the depth (on the *z* coordinate) and not the lateral position (*x,y*) so that all cells are exposed to the same concentration of nutrients and drugs within the monolayer culture. Moreover, all cells have the same rate of metabolism and volume growth at any instance with variability allowed in cell volume, rate of cell division and cell death. Monolayer ABM simulations were performed on a desktop Windows PC (Intel core I7 processor) from a Qt (Qt Company, https://www.qt.io/) graphical user interface passing parameters to a DLL built with Fortran95. The monolayer ABM uses a finite difference method to solve reaction-diffusion equations for each compound (*N*) in the medium and cells. Parameters for the cellular uptake and metabolism were fitted to the concentration-time profiles of CP-506 and its metabolites in monolayer cultures of POR-R cells using a linear (first order) cell transport and metabolism model. Terms for the extracellular instability of CP-506 and its metabolites were set at their respective values in media stability studies, and media diffusion coefficients were estimated from the support membrane diffusion coefficients described below. Experimental data describing the kinetics of CP-506 uptake under aerobic conditions was fitted by adjusting the permeability coefficients k_in_ and k_out_ for each compound. These values were fixed for anoxic experiments to allow for parameter estimation of the rate of intracellular CP-506 metabolism (k_met0_) as well as the intracellular/extracellular partitioning ratio (k_in_ and k_out_) and metabolic instability (k_met0_) for its cytotoxic metabolites.

### Pro(drug) Transport Model

Drug transport in MCLs was modelled as a one-dimensional diffusion with reaction across four consecutive compartments (stirred donor, POR-R MCL, collagen-coated teflon support membrane and stirred receiver) using a custom designed MatLab routine (Foehrenbacher et al., 2013a). Model parameters were derived by fitting Eq. 1 and Eq. 2 to the measured concentration-time profile of internal standard [^14^C]-urea, CP-506, CP-506H and CP-506M in MCL flux studies. The flux of CP-506 and its metabolites across POR-R MCLs was described using Fick’s second law with a reaction term:

$$\frac{\partial C_{\mathrm{eN}}}{\partial t}=D_{N}\frac{\partial^{2}C_{\mathrm{eN}}}{\partial x^{2}}-k_{\mathrm{lossN}}C_{\mathrm{eN}}-\varphi \left(\;k_{\mathrm{inN}}C_{\mathrm{eN}}-\;k_{\mathrm{outN}}C_{\mathrm{iN}}\right)$$ (1)

$$\frac{\partial C_{\mathrm{iN}}}{\partial t}=\;k_{\mathrm{met}0\left(N-1\right)}C_{i\left(N-1\right)}+\varphi \left(k_{\mathrm{inN}}C_{\mathrm{eN}}-\;k_{\mathrm{outN}}C_{\mathrm{iN}}\right)-k_{\mathrm{met}0N}C_{\mathrm{iN}}$$ (2)

where *C* is the concentration of the diffusing substance at position *x* and time *t* in the extracellular (*e*) or intracellular (*i*) compartment, *D* is the diffusion coefficient through either a bare support membrane (without cells) (*D*_*suo*_) or extracellular space of a POR-R MCL (*D*_*MCL*_), *k*_*loss*_ is the rate constant for first-order loss (instability) in culture medium, *k*_*in*_ and *k*_*out*_ are the rate constants for transmembrane transport and *k*_*met0*_ is the rate constant for intracellular metabolism and instability for each compound (*N*). For metabolites, *N*-1 represents the previous compound from which the current compound is produced by metabolism. Experimental data was fitted iteratively to derive a tissue diffusion coefficient (D_MCL_) and metabolic scaling factor (*φ*_*i*_) to restrain parameter estimates from the cellular PK model (K_in_, K_out_, K_met0_). The diffusion coefficient for CP-506 across the bare support membrane (D_sup_) was determined by solving Eq. 1 with a reaction term only for chemical instability. MCL thicknesses were estimated by fitting the measured [^14^C]-urea concentrations to those predicted by Eq. 1 and Eq. 2 without instability or metabolism by using the known diffusion coefficient (D_MCL_) for [^14^C]-urea in HCT-116 MCLs (Wilson et al., 2004). The diffusion coefficient (D_MCL_) for CP-506 in POR-R MCLs was then fitted from the measured concentrations in the donor and receiver compartments of supraoxic experiments by assuming one-directional diffusion in the extracellular space. The metabolic scaling factor (*φ*_*i*_) was then fitted from the measured concentrations in the donor and receiver compartment of anoxic experiments by fixing parameter estimates to the diffusion coefficient (D_MCL_) from supraoxic experiments.

### Simulations of CP-506 Diffusion and Efficacy in Spheroid Cultures

The spheroid ABM is described in further detail elsewhere (Hong et al., 2018b; Mao et al., 2018). The model employs two 3D grids and two solvers, a coarse grid represents the culture medium and is embedded with a much finer grid that represents the spheroid in a small cubic region at the bottom of the well. The spheroid grows in a specified volume and depth of unstirred medium containing dissolved oxygen. The geometry of the model is lattice-based, in which cells occupy cubic sites on a regular 3D lattice and grow in volume at a rate dependent on the local oxygen concentration. Autonomous cell mobility and cell-cell forces are not simulated. Cell motion occurs only as a result of cell division. Cells divide when the volume reaches a pre-set value (V_div_) and the resultant daughter cell occupies an adjacent empty lattice site. If a vacancy does not exist, cells are moved radially outward to create one. The oxygen concentration at the air-medium boundary is defined by the gas phase of the culture environment. As the spheroid grows and total oxygen consumption increases, concentration gradients within the culture medium and spheroid interior steepen with significantly lower concentrations within the spheroid core. When oxygen concentrations fall below a critical level (<0.15 µM), cells are tagged to die and cytolysis occurs after a further 24 h leading to the central necrosis observed in *in vitro* spheroids. Spheroid ABM simulations were performed on a desktop Windows PC (Intel core I7 processor) from a Qt (Qt Company, https://www.qt.io/) graphical user interface passing parameters to a DLL built with Fortran95. The solution of the coarser grid provides the boundary conditions for the solution of the intracellular and extracellular concentrations within the spheroid lattice. Oxygen and drug are transported by diffusion from the boundary into the spheroid interior through the intercellular space in parallel with their uptake into cells. The model allows specification of two mass transfer constants to characterise the uptake (K_in_) and efflux (K_out_) rates for each compound. Prodrug metabolism is restricted to the intracellular compartment and dependent on the local oxygen concentration. Parameter estimates for the cellular uptake and metabolism of CP-506 in monolayer cultures were used to fit clonogenic survival data to estimate the kill probability rate constant (*k*_*d*_), as described previously for PR-104A (Hong et al., 2018b). The kill probability (model 4 in the ABM program (Mao et al., 2018)) was assumed proportional to the intracellular concentration of the bioreductive metabolites (*k*_*d*_ *C*_*N*_) and was set to zero for the prodrug. The same *k*_*d*_ was assumed for each of the major metabolites (CP-506H, CP-506M and CP-506M-Cl_2_), and that the intrinsic sensitivity of the cell lines was identical with differences only in the oxygen- and POR-dependent first order rate constant for the metabolic consumption (*k*_*met0*_) of CP-506. Parameter estimates were used alongside the *in vitro* determined drug pharmacokinetic terms to predict the killing of “activator” (POR-R) and “target” (PORko-G) cells in spheroid co-cultures.

### Statistical Analysis

All statistical tests were performed using GraphPad Prism 8. Individual tests used are specified in the relevant figures.

## Results

### The Hypoxia-Dependent Metabolism and Cellular Cytotoxicity of CP-506 is Influenced by Cytochrome P450 Oxidoreductase Expression

**FIGURE 1:**
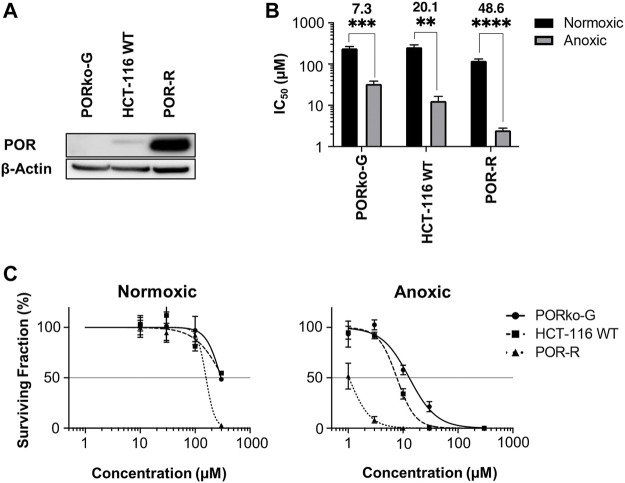
Hypoxia-dependent cellular cytotoxicity of CP-506 is influenced by POR expression. **(A)** Western blot analysis of POR expression in HCT-116 cells upon gene knockout (PORko-G) and ectopic overexpression (POR-R) as compared to the parental cell line (HCT-116 WT). **(B)** Anti-proliferative potency after exposure of monolayer cultures (700 – 1000 cells per well) to CP-506 for 4 h under normoxic (21% O_2_) and anoxic conditions (<1 ppm O_2_). IC_50_ values were determined after 96 h of drug-free proliferation as the drug concentration required to inhibit cellular proliferation by 50% of the untreated controls. Values are the mean ± SEM of triplicate wells for three independent experiments. ANOVA tests were performed using GraphPad Prism 8 (*p*-value ≤ 0.01; ***, *p*-value ≤ 0.001; ****, *p*-value ≤ 0.0001). **(C)** Clonogenic survival after exposure of monolayer cultures (4.8 x 10^5^ cells per well) to CP-506 for 4 h under normoxic (21% O_2_) and anoxic conditions (<1 ppm O_2_). IC_50_ values were interpolated from the fitted dose response curve as the concentration required to reduce the clonogenic surviving fraction to 50% of the untreated controls (shown by the reference line). Values are the mean ± SEM of duplicate wells for three independent experiments.

Given the primary role of POR in CP-506 metabolism (Van Der Wiel et al., 2021), a panel of isogenic cell lines with variable levels of POR expression was used to investigate the extravascular transport properties of CP-506 and its metabolites in an *in vitro* setting. Consistent with the genetic modifications, protein expression was elevated by 11-fold in the POR-overexpressing cell line (POR-R) relative to wild-type cells (HCT-116 WT) and was undetectable in the POR-knockout line (PORko-G) (Figure 1A). The impact of POR expression on the sensitivity of HCT-116 cells to CP-506 was examined using both an anti-proliferative (Figure 1B) and clonogenic endpoint (Figure 1C). Both assays demonstrate hypoxia-selective cellular cytotoxicity, with a marked increase in the sensitivity of HCT-116 cells to CP-506 under anoxic conditions. A 13-fold differential in anoxic sensitivity was noted between the PORko-G and POR-R cell lines by anti-proliferative assay. Aerobic IC_50_ values were 240 ± 26, 257 ± 37 and 120 ± 13 µM for the PORko-G, HCT-116 WT and POR-R cell lines, respectively (*p*-value ≤ 0.01). Values under anoxic conditions were 7.3-fold, 20.1-fold and 48.6-fold lower at 33.1 ± 5.6, 12.8 ± 3.7 and 2.5 ± 0.3 µM, respectively (p-value ≤ 0.01). Clonogenic assays showed a similar trend with a 22-fold differential in anoxic sensitivity between the PORko-G and POR-R cell lines. Clonogenic IC_50_ values under anoxia were 17.1 ± 3.4, 7.4 ± 0.5 and 0.8 ± 0.3 µM for the PORko-G, HCT-116 WT and POR-R cell lines respectively.

**FIGURE 2:**
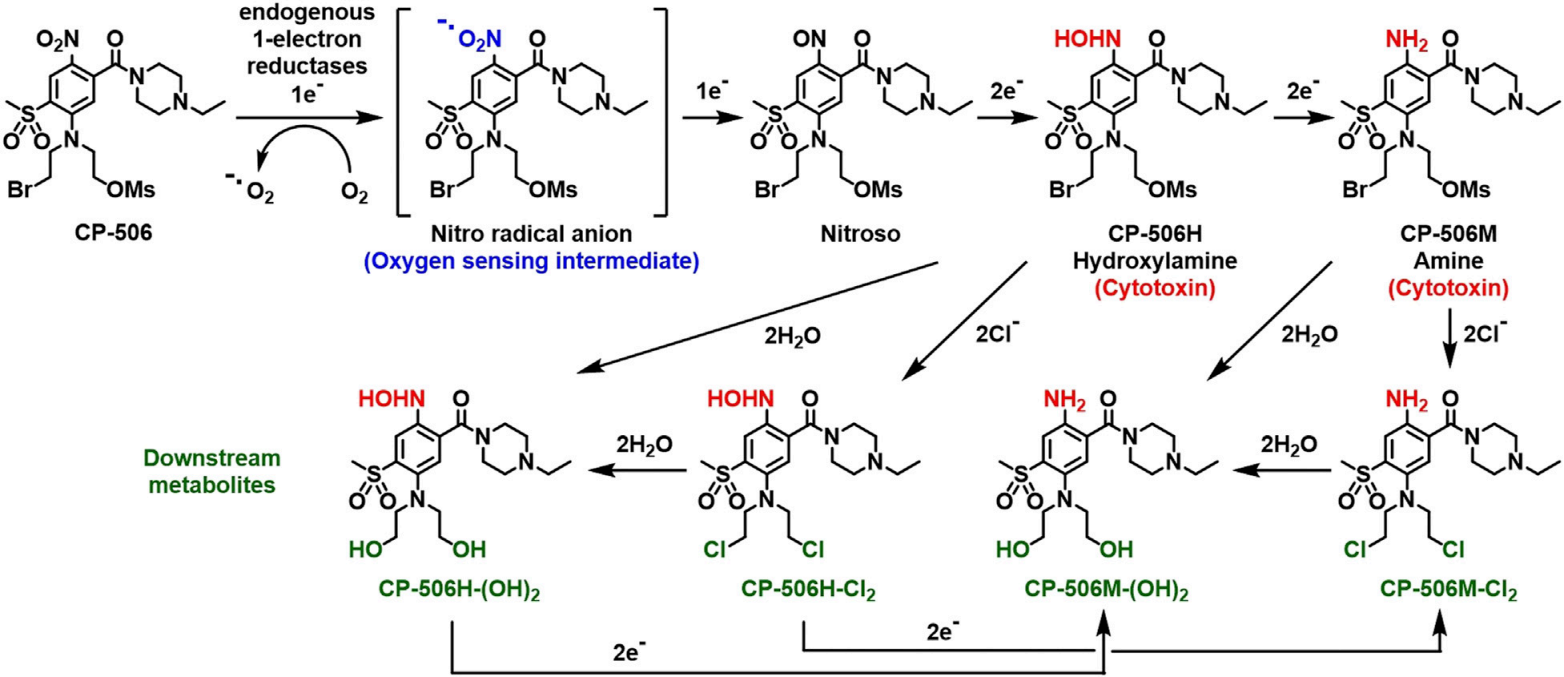
Schematic representation of the activation sequence for CP-506. CP-506 is metabolised by endogenous one-electron reductases into a nitro radical anion that is readily back-oxidised in the presence of molecular oxygen. Under hypoxia, the nitro radical anion is further metabolised to its corresponding hydroxylamine (CP-506H) and amine (CP-506M) metabolites. The major metabolites are highly reactive. Each arm of the bromomesylate mustard can react with chloride ions and/or undergo aqueous hydrolysis providing the major observed downstream metabolites, CP-506H-Cl_2_, CP-506H-(OH)_2_, CP-506M-Cl_2_, and CP-506M-(OH)_2_.

**FIGURE 3:**
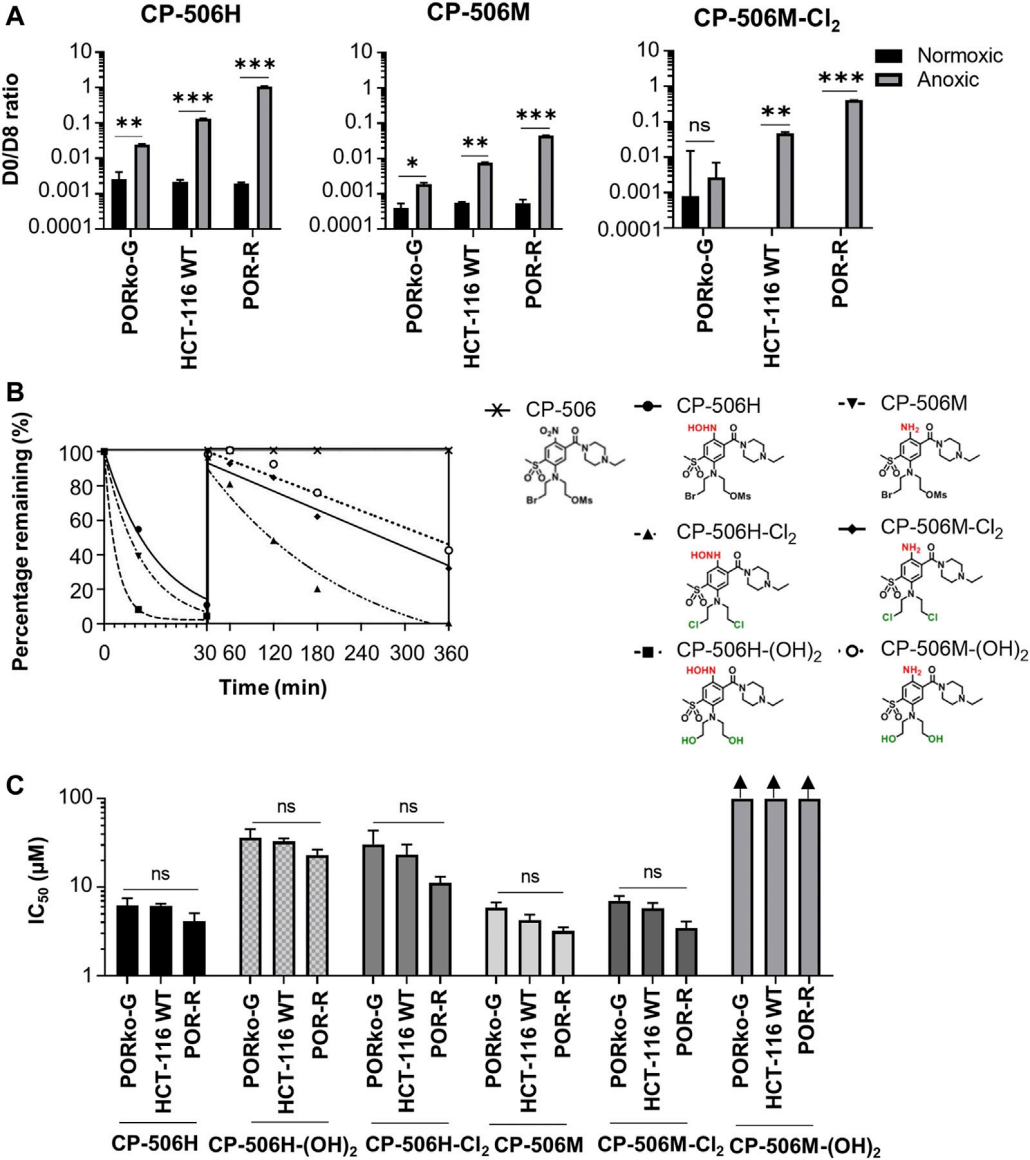
Hypoxia-dependent metabolism of CP-506 leads to the formation of a multitude of short-lived species with variable cytotoxicity. **(A)** Bioreductive metabolism of CP-506 under normoxic (21% O_2_) and anoxic conditions (<1 ppm O_2_). Concentrations of CP-506 and its metabolites (CP-506H, CP-506M and CP-506M-Cl_2_) were quantified by mass spectrometry with reference to the appropriate deuterated (D8) internal standard. Values are the mean ± SEM for three independent experiments. **(B)** Stability of CP-506 metabolites in culture medium (without cells) at 37°C. Concentrations were determined by mass spectrometry using standard curves generated from authenticated stocks. **(C)** Anti-proliferative potency after exposure of monolayer cultures to CP-506 metabolites for 5 days under normoxic conditions (21% O_2_). IC_50_ values are the mean ± SEM of duplicate wells for three independent experiments. T-tests were performed using GraphPad Prism 8 (ns, p-value > 0.05; *, *p*-value ≤ 0.05; **, *p*-value ≤ 0.01; ***, *p*-value ≤ 0.001).

The mass spectrometric detection of reduced metabolites accompanied the selective cytotoxicity of CP-506 in hypoxic cultures. Although a number of species were detected (Figure 2), the hydroxylamine CP-506H, amine CP-506M and dichloro-amine CP-506M-Cl_2_ were the major metabolites present at concentrations sufficient for comparison between cell lines. Rates of metabolite formation reflected differences in POR expression, with metabolite concentrations 5.5- (CP-506H) to 8.6-fold (CP-506M-Cl_2_) higher in POR-R cells and 4.1- (CP-506M) to 17.7-fold (CP-506M-Cl_2_) lower in PORko-G cells than HCT-116 WT cells after a 1 h treatment period (Figure 3A). Authentic standards of the six identified metabolites (Figure 2) were synthesized (see Supplementary Material) and shown to have marked differences in intrinsic stability in stirred medium at 37°C (Figure 3B). While the prodrug CP-506 showed no loss of mass balance for over 360 min in stirred medium, half-lives (T_1/2_) for the reduced metabolites were significantly shorter at 2.5, 10.5, 7.6, 141.3, 271.1 and 393.9 min for the diol-hydroxylamine CP-506H-(OH)_2_, hydroxylamine CP-506H, amine CP-506M, dichloro-hydroxylamine CP-506H-Cl_2_, dichloro-amine CP-506M-Cl_2_ and diol-amine CP-506M-(OH)_2_ respectively. Anti-proliferative IC_50_ assays were performed to estimate the relative contribution of each metabolite toward the cellular cytotoxicity of CP-506 under hypoxic conditions (Figure 3C). Both diol-containing metabolites were essentially inactive (IC_50_ > 30 μM), consistent with the absence of appropriate mustard leaving groups required for formation of the cytotoxic intermediates. The dichloro-hydroxylamine CP-506H-Cl_2_ metabolite also exhibited limited potency. The major cytotoxic metabolites (IC_50_ < 10 μM) were the hydroxylamine CP-506H, amine CP-506M, and dichloro-amine CP-506M-Cl_2_. The former two metabolites were short-lived (T_1/2_ of 7–11 min) while the latter had relatively high stability (T_1/2_ of 271 min).

### Cellular Pharmacokinetic Model for CP-506 in Tumour Tissues

**FIGURE 4:**
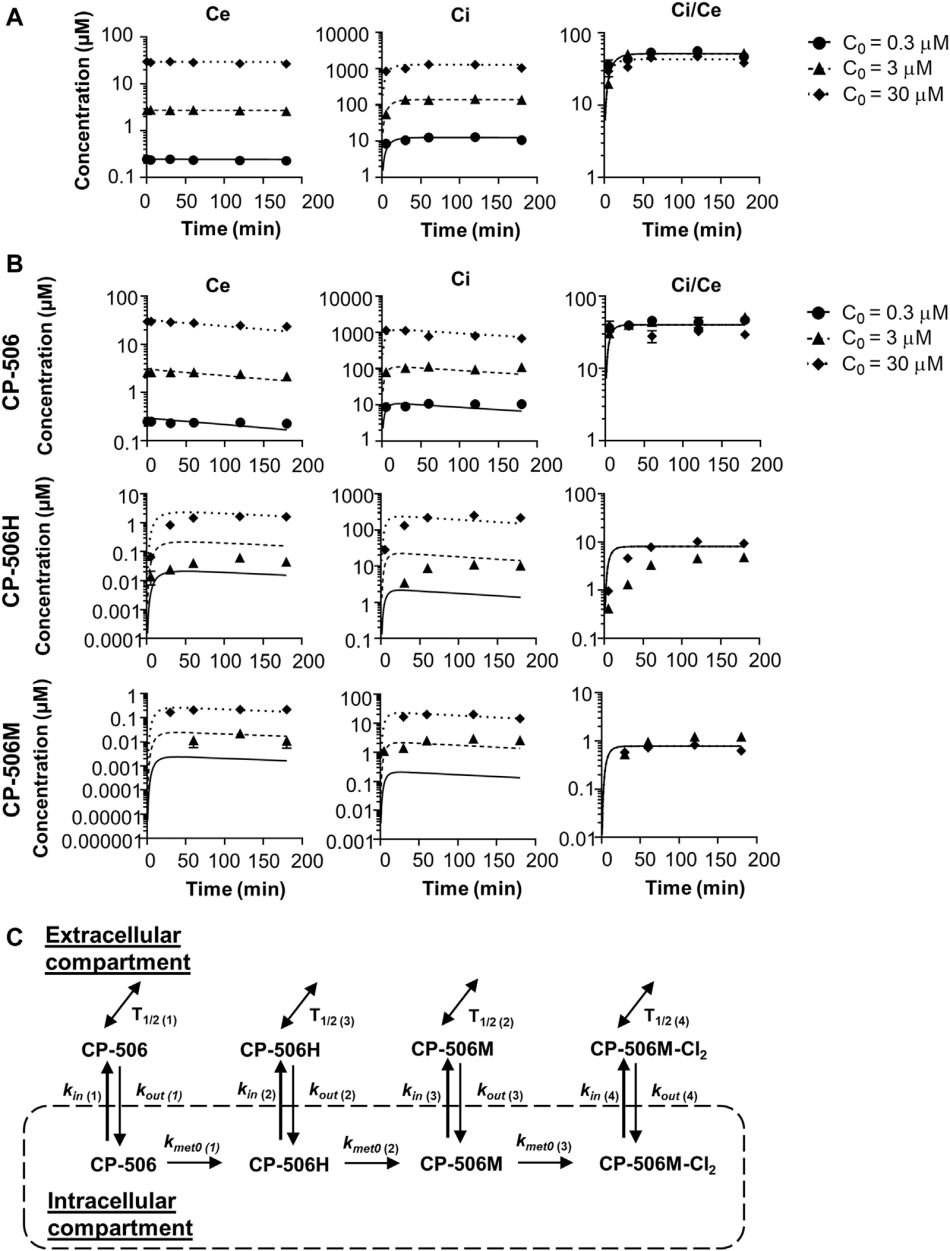
Cellular PK model of CP-506 in the tumor tissues. **(A)** Cellular uptake of CP-506 in monolayer cultures of POR-R cells after exposure to a range of initial concentrations (C_o_) under normoxic conditions (21% O_2_). Concentrations were determined in the extracellular medium and cellular extracts by mass spectrometry. Graphs show the concentration-time profile of CP-506 in the extracellular volume (C_e_), intracellular volume (C_i_) and resultant C_i_/C_e_ ratio over the experiment duration (3 h). Values represent the mean ± SEM of three independent experiments. Lines show the corresponding estimates from the cellular PK model as fitted to the experimental data. **(B)** Hypoxia-selective metabolism of CP-506 and release of CP-506 metabolites into the extracellular medium of POR-R cells under anoxic conditions (<1 ppm O_2_). Graphs show the concentration-time profile of CP-506 and its metabolites CP-506H and CP-506M in the extracellular volume (C_e_), intracellular volume (C_i_) and resultant C_i_/C_e_ ratio over the experiment duration (3 h). Data points have been censored if below the lower limits of quantification (LLOQ, 0.01 µM). Lines show the corresponding estimates from the cellular PK model. **(C)** Cellular PK model for CP-506. The concentration of CP-506 and its metabolites in the extracellular and intracellular compartments is defined by the permeability rate constants (k_in_ and k_out_). The bioreductive metabolism of CP-506 (k_met0_) is O_2_-dependent and restricted to the intracellular compartment. The stability of each compound is defined by their respective half-life (T_1/2_). Model estimates for the given parameters are provided in Supplementary Table S3.

A cellular PK model was developed based on the extracellular and intracellular concentration-time profile for CP-506 and its reduced metabolites in monolayer cultures. Experiments were performed using POR-R cells to allow for greater sensitivity in metabolite detection. Under aerobic conditions, the cellular uptake of CP-506 was described by a simple two compartment model with the loss of mass balance in the extracellular medium (C_e_) coupled with a respective increase in the intracellular concentration (C_i_) (Figure 4A). The rate of cellular uptake was fastest during the first 30 min after drug addition and plateaued thereafter. The cellular uptake of CP-506 was not concentration-dependent, with steady-state C_i_/C_e_ ratios of 50-fold across the C_o_ range of 0.3–30 µM initial concentrations. No net metabolism nor intrinsic instability were observed under aerobic conditions. In anoxic experiments, the cellular uptake of CP-506 was accompanied by the appearance of metabolites CP-506H and CP-506M in the intracellular compartment (Figure 4B). The loss of mass balance was reiterated in the lower steady-state C_i_/C_e_ ratio of 40-fold. The formed metabolites were released from the cell of origin into the extracellular medium indicating bystander potential. However, the mass balance was dominated by the intracellular fraction indicating that the cellular efflux of these metabolites is a passive process. A first order kinetic model describing the cellular uptake and metabolism of CP-506 (Figure 4C) was fitted to the *in vitro* data with excellent correlation between the experimental data and model-predicted fits (R^2^ = 0.9596) (lines in Figure 4B). The model assumed that the high intracellular concentrations of CP-506 are driven by its large cell uptake factor, and that metabolic loss of CP-506 is O_2_-dependent and restricted to the intracellular compartment. Once formed, the downstream metabolites can diffuse freely between the two compartments. The stability varies between compounds with the half-life (T_1/2_) of CP-506 significantly longer than that of its metabolites. The rate of CP-506 metabolism and metabolite uptake is higher than the rate of cellular efflux resulting in the high intracellular concentrations observed under anoxic conditions. Model estimates of the uptake and metabolism parameters for CP-506 and its metabolites are provided in Supplementary Table S3. Cell uptake studies considered only the major metabolites (CP-506H and CP-506M) due to the retrospective identification and synthesis of the downstream metabolites and deuterated internal standards thereof. For inclusion in the cellular PK model, the rate constants for the cellular uptake, cellular efflux and extracellular instability of CP-506M-Cl_2_ were fixed to the fitted values for the stable downstream metabolites of PR-104A (termed “metabolite 2”) (Foehrenbacher et al., 2013a; Hong et al., 2018b). Parameter estimates were later refined based on the accrued stability and MCL transport data for spheroid simulations as described below.

### Extravascular Transport Model for CP-506 in Tumour Tissues

**FIGURE 5:**
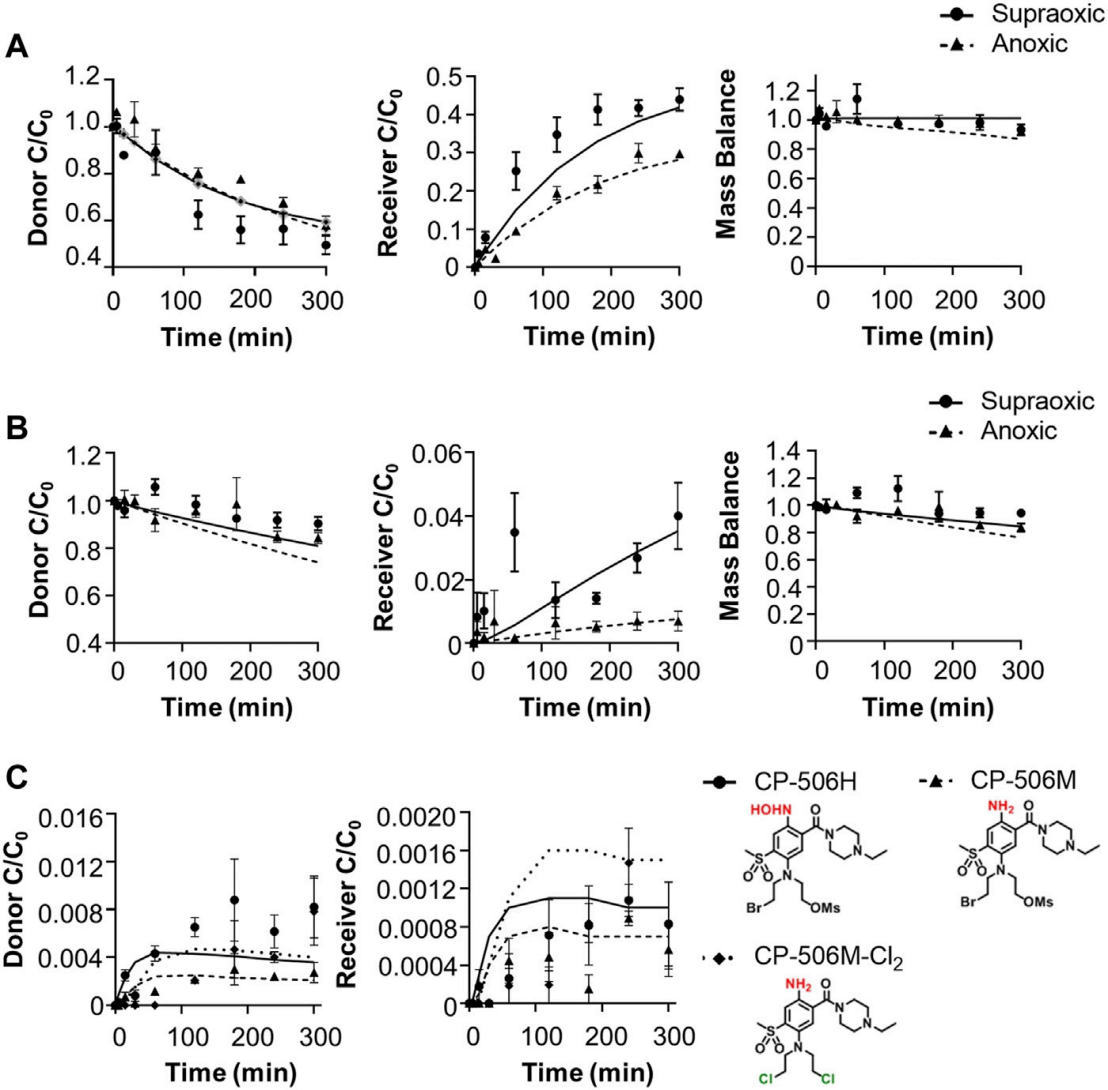
Extravascular transport of CP-506 and its formed metabolites (CP-506H, CP-506M and CP-506M-Cl_2_) across support membranes with or without a MCL culture. Flux was initiated by the addition of CP-506 (C_o_ = 17.4 ± 1.3 µM) and internal standard [^14^C]-urea into the donor compartment of diffusion chambers maintained under supraoxic (95% O_2_, 5% CO_2_) and anoxic (5% CO_2_, bal N_2_) conditions. The concentration-time profile of CP-506 in the donor and receiver compartments of diffusion chambers was determined by mass spectrometry. Mass balance was calculated as the sum of the donor and receiver compartments. Lines are the concentrations predicted from the developed (pro)drug transport model. Values are mean ± SEM of three independent diffusion chambers. **(A)** Concentration-time profile of CP-506 across bare support membranes (without cells) maintained under supraoxic and anoxic conditions. **(B)** Concentration-time profile of CP-506 across POR-R MCLs maintained under supraoxic and anoxic conditions. **(C)** Concentration-time profile for the formed metabolites of CP-506 (CP-506H, CP-506M and CP-506M-Cl_2_) in POR-R MCLs under anoxic conditions.

The cellular uptake and metabolism parameters from monolayer assays were scaled to tissue-like densities based on the concentration-time profile of CP-506 and its formed metabolites in the donor and receiver compartment of diffusion chambers containing multicellular layers maintained under supraoxic (95% O_2_, 5% CO_2_) and anoxic conditions (5% CO_2_, bal N_2_). Initial experiments were performed using collagen-coated inserts without cells to determine the chemical stability and diffusion coefficient of CP-506 across the bare support membrane (D_sup_) (Figure 5A). Irrespective of oxygen tension, the extravascular transport of CP-506 across the bare support membrane was described using a simple Fickian diffusion model without loss of mass balance between the media compartments of the diffusion chambers (donor, receiver). Differences in stirring rate resulted in a lower D_sup_ for anoxic experiments ((0.717 ± 0.01) x 10^−6^ cm^2^ s^−1^) compared to supraoxic experiments ((1.32 ± 0.19) x 10^−6^ cm^2^ s^−1^), however this discrepancy was accounted for in D_MCL_ calculations. MCL cultures were established from POR-R cells to allow for greater sensitivity in metabolite detection. The predicted thickness of the MCL cultures (L_MCL_) was 101.7 ± 6.9 µm, as estimated from the acquired flux data and established D_MCL_ (3.67 x 10^−7^ cm^2^ s^−1^, (Wilson et al., 2004)) for internal standard [^14^C]-urea. Drug transport in the MCL was modelled as a one-dimensional diffusion with reaction in the extracellular and intracellular compartments, with the integrated reaction term used to describe the loss of mass balance due to cellular uptake and metabolism. The cellular uptake of CP-506 slowed extravascular transport across the MCL, as indicated by the estimated D_MCL_ of (1.93 ± 0.011) x 10^−7^ cm^2^s^−1^ for supraoxic experiments (Figure 5B). The rate of diffusion was further reduced by reductive metabolism with its major metabolites detected in both the donor and receiver compartments of anoxic experiments (Figure 5C). A metabolic scaling factor (φ_i_) of 0.3 was derived to scale the metabolic parameters from the previous monolayer-based assays. A summary of the determined extravascular transport parameters for CP-506 across POR-R MCLs is provided in Supplementary Table S4.

### Bystander Potential of CP-506 in Spheroid Co-cultures

**FIGURE 6:**
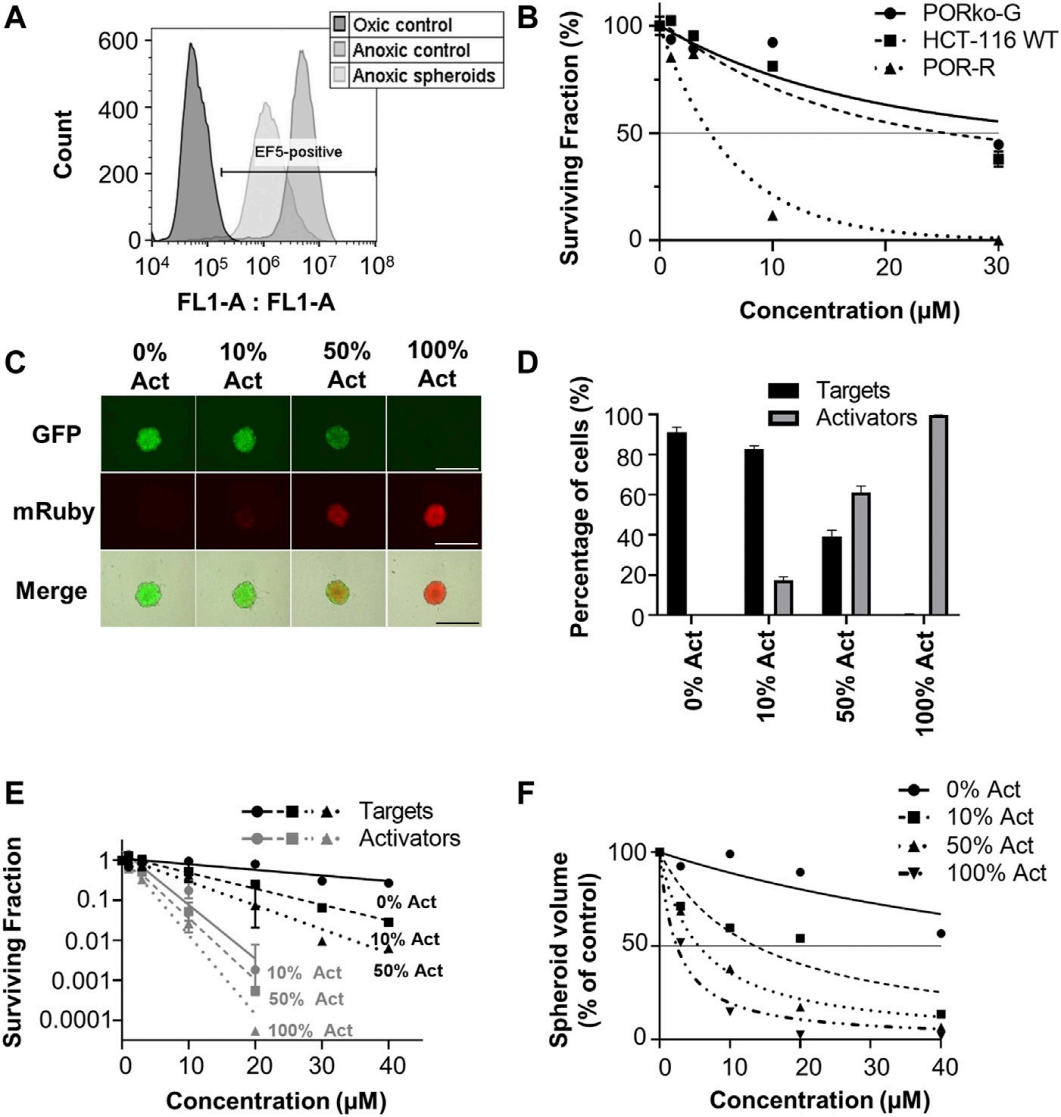
Bystander efficiency of CP-506 in spheroid co-cultures. Multicellular spheroids were established by seeding differing proportions of target (PORko-G) and activator cells (POR-R) and growing for 4 days in ultra-low attachment, round bottom 96-well plates. **(A)** Flow cytometric analysis of EF5 binding upon the enzymatic dissociation of anoxia pre-equilibrated POR-R spheroids. The hypoxic population was defined by the hypoxia-selective metabolism and binding of EF5 in monolayer controls. **(B)** Clonogenic survival after exposure of spheroid cultures to CP-506 for 4 h under anoxic conditions (<1 ppm O_2_). IC_50_ values were interpolated from the fitted dose response curve as the concentration required to reduce the plating efficiency to 50% of the untreated controls (shown by the reference line). Values are the mean ± SEM for three independent experiments. **(C)** Fluorescent microscope images of representative spheroids showing target (GFP-positive) and activator cells (mRuby-positive) at the anticipated proportions based on their initial seeding densities. Images were acquired at 4 X objective using a JuliStage Real-Time Cell History Recorder (NanoEnTek, Seoul, Korea). Scale bar indicates 1 mm. **(D)** Flow cytometric detection of target and activator cells in spheroid co-cultures based on GFP expression. The parental cell line (HCT-116 WT) was used as a negative control. Values are the mean ± SEM for three independent experiments. **(E)** Clonogenic survival of target and activator cells (Act) in spheroid co-cultures treated with CP-506 for 4 h under anoxic conditions (<1 ppm O_2_). Spheroids were then enzymatically dissociated and plated in parallel in media supplemented with 2 µM puromycin or 1 mg/ml geneticin to select for activator and target cells respectively. Values are the mean ± SEM for three independent experiments. **(F)** Growth delay after exposure of spheroid co-cultures to CP-506 for 4 h under anoxic conditions (<1 ppm O_2_). Spheroid volumes were quantified at endpoint (day 14 post-treatment) using a previously described method of image analysis (Mao et al., 2018). Values are the mean ± SEM for three independent experiments.

Predictions based on the tissue pharmacokinetics of CP-506 in experimental model systems were compared to determinations of clonogenic cell survival and growth delays in spheroid co-cultures. Experiments were performed under strict anoxia to eliminate oxygen gradients to ensure that the established concentration gradients of CP-506 and its reduced metabolites reflect their respective pharmacokinetic properties rather than metabolic potential due to oxygen availability. This notion was confirmed by the flow cytometric detection of EF5 adducts (<1 µM O_2_) upon the enzymatic dissociation of anoxic spheroids (Figure 6A). Preliminary dose ranging experiments identified a 22-fold differential in anoxic sensitivity between PORko-G and POR-R spheroids by clonogenic endpoint reflecting the intrinsic differences in metabolic potential between the cell lines (Figure 6B).

Spheroid co-cultures were established by seeding “activator” POR-R cells and “target” PORko-G cells at differing proportions growing for 4 days in ultra-low attachment, round bottom 96-well plates. At this point, fluorescent imaging showed intimate mixtures of red and green fluorescent cells in proportions broadly consistent with the respective seeding densities of activator and target cells (Figure 6C). A flow cytometric analysis of representative spheroids showed that activator cells were overrepresented by 5–10% due to the longer doubling time of the target cells (21.7 ± 0.04 vs. 22.95 ± 0.38 h, respectively) (Figure 6D).

Established spheroids were exposed to CP-506 for 4 h under anoxic conditions. Treated spheroids were then enzymatically dissociated for a clonogenic endpoint or tracked using an image analysis method of volume determination. Clonogenic survival assays showed increased killing of both target and activator cells with the proportion of activator cells in the co-culture (Figure 6E). Growth delay assays illustrated a similar trend with an increase in the proportion of activator cells leading to greater spheroid growth inhibition at each concentration relative to the DMSO-only control (Figure 6F). IC_50_ values for inhibition of spheroid growth were >40 µM, 13.6 µM, 5.6 µM and 2.4 µM for spheroid co-cultures comprised of 0%, 10%, 50% and 100% activator cells respectively.

**FIGURE 7:**
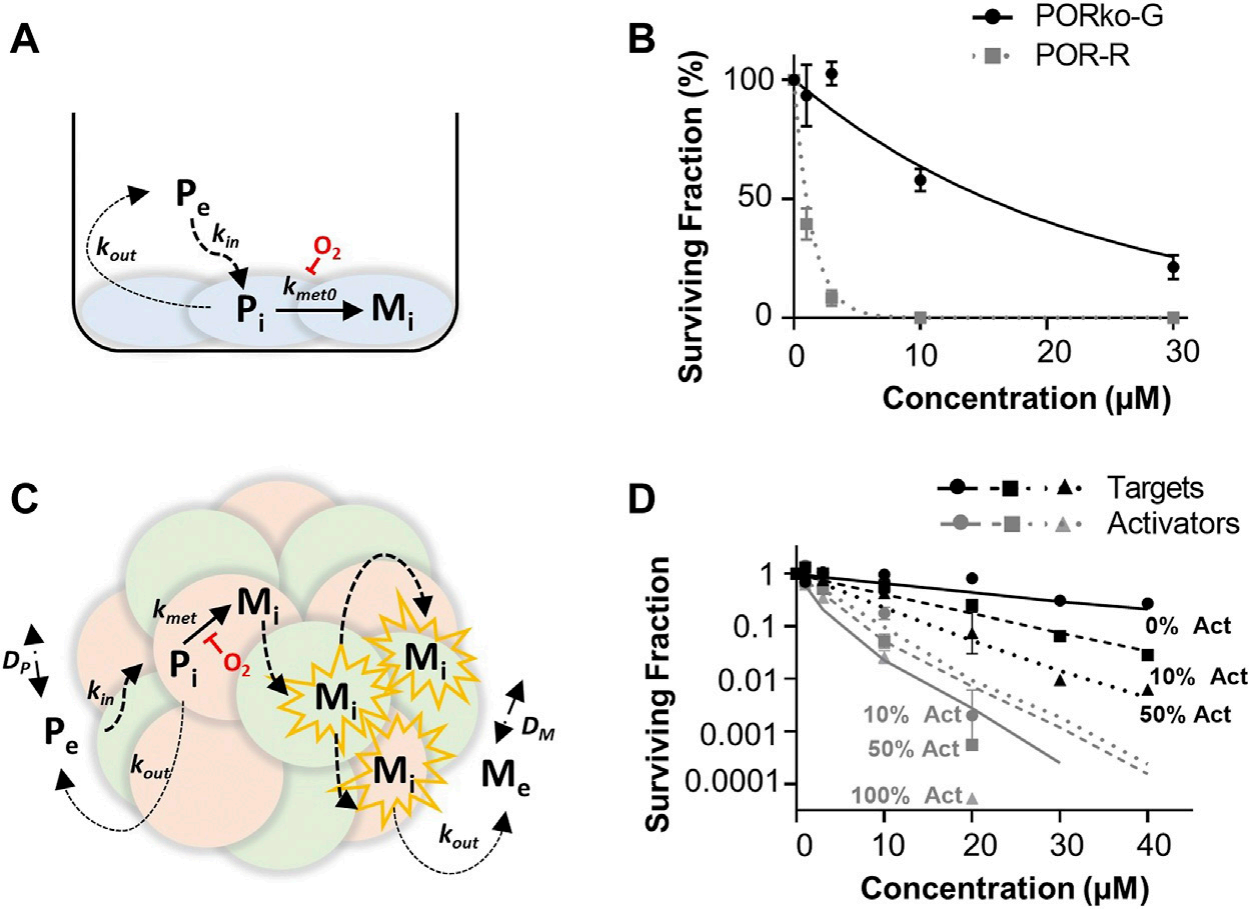
ABM simulations of clonogenic cell survival in monolayer and spheroid cultures following CP-506 treatment. **(A)** Schematic representation of the cellular uptake, metabolism and diffusion of CP-506 in monolayer cultures. Exchange of the extracellular prodrug (P_e_) and intracellular prodrug (P_i_) between cell membranes is defined by permeability rate constants k_in_ and k_out_ with metabolism of P_i_ to intracellular metabolites (M_i_) defined by the first order rate constant k_met0_ and is strictly oxygen-dependent. **(B)** Model predictions (lines) compared to experimental determinations (symbols) of clonogenic survival in monolayer cultures of target (PORko-G) and activator (POR-R) cells exposed to CP-506 for 4 h under anoxic conditions (<1 ppm O_2_) determined from the monolayer ABM by fitting k_d_ for POR-R cells and the k_met0_ parameter for PORko-G cells (data redrawn from Figure 2C). **(C)** Schematic representation of the cellular uptake, metabolism and diffusion of CP-506 in spheroid co-cultures. Diffusion of extracellular prodrug (P_e_) and intracellular prodrug (P_i_) across cell membranes is defined by permeability rate constants k_in_ and k_out_. P_i_ is metabolized to intracellular metabolites (M_i_) in activator cells (red) defined by a first order rate constant k_met0_ which is oxygen-dependent. M_i_ can diffuse to nearby target cells (green) by the extracellular route to elicit bystander killing and can diffuse out into the extracellular compartment, M_e_. In the extracellular compartment P_e_ and M_e_ can diffuse as defined by their diffusion coefficients D_P_ and D_M_ respectively. **(D)** Model predictions (lines) compared to experimental determinations (symbols) of clonogenic survival in spheroid co-cultures comprised of differing proportions of activator (act) and target cells (data re-drawn from Figure 6E).

A summary of the parameters used for ABM simulations is provided in Table 1. Here, the *in vitro* determined terms for the cellular uptake, metabolism and transport of CP-506 were used to simulate the killing of activator and target cells in anoxic spheroids. The clonogenic cell survival data from monolayer-based assays was used to estimate the kill probability parameter (k_d_) and was well-fitted with the monolayer ABM (*R* ^2^ = 0.9685; Figure 7A). This assumed uniform solute concentrations and equipotent metabolites (same k_d_) with a lower anoxic first-rate constant for metabolic consumption (k_met0_) fitted for target compared to activator cells (0.0019 min^−1^ vs. 0.12 min^−1^ respectively) (Figure 7B). However, a higher k_d_ was required to model the experimental data from spheroid co-cultures (0.01 vs. 0.0256 respectively). The spheroid ABM assumed that diffusion occurs through the unstirred medium and extracellular spaces with the metabolic activation of CP-506 restricted to the intracellular space (Figure 7C). CP-506 and its metabolites can exchange across the plasma membrane with the reduced metabolites sufficiently stable to diffuse from the cell of origin. Simulated spheroids were exposed to CP-506 for 4 h under anoxic conditions with oxygen gradients restored upon treatment withdrawal. Best efforts recapitulated trends in target cell killing (*R* ^2^ = 0.9733) but slightly underestimated activator cell killing (*R* ^2^ = 0.7703; Figure 7D).

**TABLE 1:** CP-506 PK/PD parameters used for ABM simulations.

Parameter (unit)	Description	Estimate			
		CP-506	CP-506H	CP-506M	CP-506M-Cl_2_
*D*_*s*_ (cm^2^s^−1^)^a^	Diffusion coefficient in spheroid	1.93 × 10^−7^ ± 1.8 × 10^−8^	1.93 × 10^−7^ ± 1.8 × 10^−8^	1.93 × 10^−7^ ± 1.8 × 10^−8^	1.93 × 10^−7^ ± 1.8 × 10^−8^
*D*_*M*_ (cm^2^s^−1^)^b^	Diffusion coefficient in medium	1.32 × 10^−6^ ± 0.19 × 10^−6^	1.32 × 10^−6^ ± 0.19 × 10^−6^	1.32 × 10^−6^ ± 0.19 × 10^−6^	1.32 × 10^−6^ ± 0.19 × 10^−6^
*k*_*in*_ (min^−1^)^c^	Rate constant for transfer from the extracellular to intracellular volume	3.7	0.9	0.9	0.9^d^
*k*_*out*_ (min^−1^)^c^	Rate constant for transfer from the intracellular to extracellular volume	0.06	0.25	0.4	0.3^d^
Half-life (h)^e^	Time required to reduce the concentration of drug to half of its initial value	30	0.18	0.13	4.52
Activator *k*_*met0*_ (min^−1^)^c^	Rate constant for the maximum rate of metabolism in POR-R cells under anoxia	0.12	0.09	0.1	0.1^d^
Target *k*_*met0*_ (min^−1^)^f^	Rate constant for the maximum rate of metabolism in PORko-G cells under anoxia	0.0019	0.09	0.1	0.1^d^
*k*_*d M*_ ^f^	Kill probability rate constant in monolayer cultures	0	0.01	0.01	0.01
*k*_*d S*_ ^f^	Kill probability rate constant in spheroid cultures	0	0.0256	0.0256	0.0256

a D_MCL_ fitted to the concentration-time profile of CP-506 in the donor and receiver compartment of diffusion chambers bearing POR-R MCLs maintained under supraoxic conditions. Parameter estimates were assumed equivalent for the active metabolites due to their similar physiochemical properties – Refer to Figure 5.

b D_sup_ fitted to the concentration-time profile of CP-506 in the donor and receiver compartment of diffusion chambers bearing bare support membranes maintained under supraoxic conditions. Parameter estimates were assumed equivalent for the active metabolites due to their similar physiochemical properties – Refer to Figure 5.

c Parameters estimated from the extracellular/intracellular partitioning of CP-506 and its active metabolites in monolayer cultures maintained under supraoxic or anoxic conditions – Refer to Figure 4.

d Extracellular/intracellular partitioning parameters of CP-506M-Cl_2_ were assumed equivalent to the measured metabolites or derived from published estimates for PR-104A metabolites (Foehrenbacher et al., 2013a; Hong et al., 2018b).

e Stability of CP-506 and its active metabolites in stirred culture medium at 37°C – Refer to Figure 3.

f Parameters estimated from the clonogenic survival of monolayer or spheroid cultures following exposure to CP-506 under anoxic conditions – Refer to Figure 7.

**FIGURE 8:**
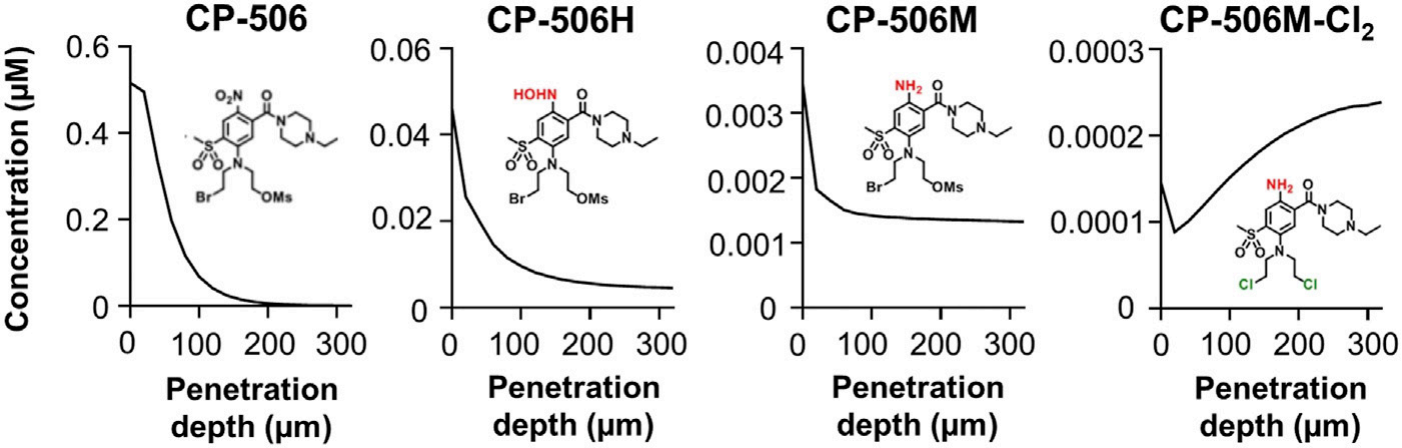
ABM simulations of the intracellular concentration gradients of CP-506 and its reduced metabolites in a transverse section of a multicellular spheroid. Model predictions of the intracellular concentration gradients of CP-506 and its reduced metabolites CP-506H, CP-506M and CP-506M-Cl_2_ as a function of penetration depth in a spheroid co-culture comprised of 50% activators after exposure to 20 µM CP-506 for 4 h under anoxic conditions (<1 ppm O_2_).

To understand how its pharmacological properties may impact tissue distribution in in vivo tumours, spatial gradients of CP-506 and its reduced metabolites were modelled in a transverse section of a representative spheroid comprised of 50% activators (Figure 8). Simulations show intracellular concentrations as a function of penetration depth after a 4 h exposure under anoxic conditions. The model predicts a rapid decline in the intracellular concentration of CP-506 from the spheroid periphery toward the spheroid core (613-fold range). Concentrations are negligible beyond a penetration depth of 190 µm. Concentrations of the formed metabolites CP-506H and CP-506M largely reflect the spatial gradient of CP-506 within the spheroid, with concentrations naturally higher in the peripheral cells. The decline in the intracellular concentration is however less pronounced compared to CP-506, with concentrations in the innermost cells only 3- (CP-506M) to 10-fold (CP-506H) lower than that of the peripheral cells. Intracellular concentrations of the downstream metabolite CP-506M-Cl_2_ mirror the spatial gradients of CP-506H and CP-506M, with concentrations markedly higher in the spheroid core. Collectively, these findings indicate that the reduced metabolites of CP-506 are sufficiently stable to diffuse from their hypoxic activating cells to neighbouring populations to potentiate cell killing by means of a bystander effect.

## Discussion

This study demonstrates that CP-506 exhibits favourable tissue pharmacokinetic properties, with a high cell uptake factor, maximal activation under extreme hypoxia, long half-life and good aqueous stability; all properties consistent with the observed anti-tumour efficacy of CP-506 (Van Der Wiel et al., 2021). These optimal properties are comparable or superior to the first generation analogue PR-104A (Patterson et al., 2007; Patel et al., 2011) but without the potential for off-target aerobic activation by AKR1C3 in normal tissues. The clinical development of PR-104 was halted due to dose-limiting myelotoxicity at suboptimal plasma exposures (Jameson et al., 2010; Abou-Alfa et al., 2011; Mckeage et al., 2012) with AKR1C3 expression in human bone marrow progenitor cells implicated as the probable cause (Birtwistle et al., 2009; Guise et al., 2010; Van Der Wiel et al., 2021). CP-506 thus represents an optimised PR-104 analogue designed to undergo metabolic activation exclusively under hypoxic conditions (Van Der Wiel et al., 2021).

Here, we employ a series of novel experimental systems to solve reaction-diffusion equations to define parameters for the cellular uptake, metabolism and diffusion of CP-506 and its active metabolites (Figure 2) in tumour tissue. Model predictions were validated by comparing theoretical predictions to experimental determinations of clonogenic cell survival in spheroid co-cultures comprised of varying proportions of metabolically competent “activator” and metabolically defective “target” cells.

The cellular uptake of CP-506 in monolayer cultures was described by a first order kinetic model with a constant cell uptake (C_i_/C_e_) ratio of 50-fold (Figure 4A). The physiochemical properties of CP-506 imply that the high affinity cellular uptake is due to lysosomal sequestration reflecting ionization of the piperazine nitrogen (pKb = 7.79). The developed prodrug transport model suggests that the high cell uptake factor is offset by the intrinsic stability of CP-506 in the absence of reductive metabolism (Figure 5B), where the extended half-life affords the long equilibrium time required for delivery of effective concentrations to distal, metabolically active hypoxic cells (<1 µM O_2_) (Van Der Wiel et al., 2021).

The reliance on severe hypoxia (<1 µM O_2_) for CP-506 metabolism and cytotoxicity (Van Der Wiel et al., 2021) ensures selective activation in tumour tissues. A number of flavoenzymes have been implicated in the nitroreduction of CP-506 in an *in vitro* setting (Van Der Wiel et al., 2021), including POR which is a major enzyme responsible for the hypoxia-selective metabolism of PR-104A in cancer cell lines (Guise et al., 2007; Guise et al., 2012). Here, we reaffirm the role of POR in CP-506 metabolism using paired cell lines with variable levels of protein expression. Forced overexpression of soluble POR heightened protein expression levels by 11-fold with corresponding increase in the rate of CP-506 metabolite formation (Figure 3A) and cellular cytotoxicity (Figure 1B) under hypoxic conditions. POR gene knockdown had the converse effect, with metabolite production suppressed up to 151-fold (CP-506M-Cl_2_, Figure 3A) relative to the POR-overexpressing cell line resulting in a 20-fold differential in anoxic sensitivity by clonogenic endpoint in both monolayer (Figure 1C) and spheroid-based assays (Figure 6B).

The hypoxia-selective metabolism of CP-506 leads to the formation of a series of reactive cytotoxic species (Figure 2). By direct exogenous exposure of cell monolayers to each authentic metabolite, we identify the amine CP-506M, hydroxylamine CP-506H and dichloro-amine CP-506M-Cl_2_ as the major cytotoxic metabolites (Figure 3). Our spheroid-based assays provide the first direct evidence of a bystander effect, with reduced growth kinetics (Figure 6F) and increased target and activator cell killing (Figure 6E) correlating with the proportion of activator cells in the co-culture. These findings are consistent with metabolite redistribution via efflux of the formed metabolites from metabolically enhanced “activator” cells followed by rapid uptake into neighbouring POR-null “target” cell populations leading to widespread cell killing. Moreover, heightened “activator” cell killing with escalating proportions of activators indicates that metabolic consumption of CP-506 does not impede its extravascular transport to the most distal regions of the tumour tissue resulting in minimal spatial heterogeneity in metabolite distribution. This interpretation is supported by the appearance of the active metabolites in the media compartments of our experimental model systems (Figure 4, Figure 5), although the attained parameters slightly underestimate activator cell killing (Figure 7D). This implies that the extravascular transport properties of CP-506 are likely superior to that anticipated by our experimental model systems. It may also indicate missing variables in our bystander model, analogous to the implied requirement for metabolites with low potency but high stability for PR-104A (Foehrenbacher et al., 2013a). Despite these caveats, our simulations predict a striking bystander efficiency at tissue-like densities (Figure 8). Although reaction (cellular uptake and metabolism) within the tumour tissue completely depletes intracellular concentrations of CP-506 beyond a penetration depth of 190 µm, concentrations of the formed metabolites were fairly homogenous throughout the simulated spheroid. In fact, intracellular concentrations of the major metabolites were only 3- (CP-506M) to 10-fold (CP-506H) lower at a penetration depth of 300 µm, essentially compensating for the CP-506 concentration gradient.

CP-506 is metabolically converted under hypoxic conditions into a series of short-lived reduced metabolites (Figure 2), but the relative contribution of each toward the bystander efficiency of CP-506 is not yet known. In principle, the bystander potential of each metabolite is dependent on multiple factors including its rate of intracellular production, stability, potency and membrane permeability. Of the metabolites examined, CP-506H-Cl_2_ and CP-506M-Cl_2_ demonstrated at least a 10-fold longer half-life than CP-506H or CP-506M (Figure 3B), reflecting the slower leaving group efficiency of the *bis*-chloro-mustard metabolites. Analogous to the dichloro metabolites of PR-104A (Hong et al., 2018b), the dichloro-hydroxylamine CP-506H-Cl_2_ and dichloro-amine CP-506M-Cl_2_ are predicted to be approximately 10-fold more lipophilic than their corresponding Br/OMs mustard metabolites CP-506H and CP-506M, indicating that they will more readily permeate membranes and exit the cell of origin. Notably, the stability of CP-506H-Cl_2_ was relatively poor in comparison to CP-506M-Cl_2_, reflecting the tendency of the hydroxylamine moiety to undergo further reduction to its amine derivative, CP-506M-Cl_2_. In an analogous manner, the diol-hydroxylamine CP-506H-(OH)_2_ was undetectable in anoxic cultures indicating rapid conversion to its six-electron reduction product CP-506M-(OH)_2_ (Figure 3B). Despite the long half-life of CP-506M-(OH)_2_, the cell line panel was not sensitive to concentrations up to 100 µM (Figure 3C), consistent with the hydroxyl moieties failing to act as leaving groups (and the hydrophilic nature of the metabolite). This identifies CP-506M-(OH)_2_ as an inert terminal metabolite. Collectively these data also indicate the reduction from CP-506H-Cl_2_ to CP-506M-Cl_2_ proceeds faster than its hydrolysis to CP-506H-(OH)_2_. Taken together, the superior physicochemical properties of CP-506M-Cl_2_ are consistent with the simulated accumulation of CP-506M-Cl_2_ in the spheroid core and identify it as the major bystander mediator of CP-506, perhaps with CP-506H and CP-506M playing minor secondary roles.

While the concentration-dependence of “activator” and “target” cell killing with CP-506 is comparable to previous determinations for PR-104A (Hong et al., 2018b), it is important to note any difference in plasma pharmacokinetics are not accounted for in the current modelling paradigm. Although PR-104A showed favourable activity in murine xenograft models (Patterson et al., 2007), safe human exposure levels were only 10–29% of those observed in mice (Guise et al., 2010), most likely due to off-target aerobic activation of PR-104A by human AKR1C3 in myeloid progenitor cells. The resistance of CP-506 to AKR1C3 bioactivation predicts for significantly higher plasma exposures in human trial without dose-limiting myelotoxicity. When coupled with the favourable extravascular transport properties of CP-506 and its bystander metabolite(s), as described in this study, marked single agent activity would be anticipated. Substantial anti-tumour activity of CP-506 has indeed been reported using clinically-relevant doses and schedules in murine xenograft models of diverse cancer types with variable hypoxic fractions reflecting the superior physicochemical properties and murine pharmacokinetics of CP-506 (Van Der Wiel et al., 2021). Current research efforts are exploring this further using a well-validated *in silico* spatially-resolved pharmacokinetic/pharmacodynamic modelling approach (Foehrenbacher et al., 2013a), where experimental inputs are used to model the spatial gradients of a prodrug and its cytotoxic effectors with respect to molecular oxygen in a representative mapped tumour microvascular network.

Overall, this study uses a series of novel experimental systems along with computational modelling to investigate the extravascular transport properties and bystander efficiency of nitrogen mustard prodrug CP-506 and its active metabolites in the tumour tissues. We show that CP-506 possesses favourable tissue pharmacokinetic properties for anti-tumour activity including a long half-life, high cell uptake factor, tissue diffusion, selective activation under severe hypoxia and a substantial bystander potential. Our findings endorse the ongoing clinical development of CP-506 (NCT04954599) for use in the treatment of patients with advanced solid malignancies.

## Data Availability

The raw data supporting the conclusion of this article will be made available by the authors, without undue reservation.
