# Supplementary material for: Tissue Pharmacokinetic Properties and Bystander Potential of Hypoxia-Activated Prodrug CP-506 by Agent-Based Modelling
Source: Front Pharmacol. 2022 Feb 8;13:803602. doi: 10.3389/fphar.2022.803602 (PMC8861431; doi:10.3389/fphar.2022.803602)
Supplement: Supplementary file 1 [file DataSheet1.docx]

# SUPPLEMENTARY METHODS

## Synthesis of CP-506H-(OH)_2_, CP-506M-(OH)_2_, CP-506H-Cl_2_, CP-506M-Cl_2_ and their respective D8 standards

CP-506H-(OH)_2_ and CP-506M-(OH)_2_ were synthesized according to the route shown in Supplementary Scheme 1. Briefly, fluorobenzene **1** (1) was reacted with diethanolamine in DMSO at room temperature to give diol **2**. Hydrogenation (40 psi) of diol **2** over Raney®-Nickel (activated catalyst, 50% slurry in water) gave the hydroxylamine CP-506H-(OH)_2_. Alternately, hydrogenation (40 psi) of diol **2** over platinum on carbon (5 wt. %) gave the amine CP-506M-(OH)_2_.

**Supplementary Scheme 1.** Synthetic route to CP-506H-(OH)_2_ and CP-506M-(OH)_2_

CP-506H-Cl_2_ and CP-506M-Cl_2_ were synthesised according to the route shown in Supplementary Scheme 2. Briefly, reaction of diol **2** with methanesulfonyl chloride and triethylamine in dichloromethane at 0 ^o^C gave the bis-mesylate **3**. Lithium chloride mediated displacement of the mesylate groups of bis-mesylate **3** in acetone at room temperature gave the bis-chloro mustard **4**. Hydrogenation (40 psi) of mustard **4** over Raney®-Nickel (activated catalyst, 50% slurry in water) gave the hydroxylamine CP-506H-Cl_2_. Alternately, hydrogenation (40 psi) of mustard **4** over palladium on carbon (5 wt. %) gave the amine CP-506M-Cl_2_.

**Supplementary Scheme 2.** Synthetic route to CP-506H-Cl_2_ and CP-506M-Cl_2_

CP-506H-(OH)_2_-8D and CP-506M-(OH)_2_-8D, the octa-deuterated standards of the parent compounds, were synthesised according to the route shown in Supplementary Scheme 3. Briefly, fluorobenzene **1** (1) was reacted with diethanolamine-d8 in DMSO at room temperature to give diol-d8 **5**. Hydrogenation (40 psi) of diol-d8 **5** over Raney®-Nickel (activated catalyst, 50% slurry in water) gave the hydroxylamine CP-506H-(OH)_2_-8D. Alternately, hydrogenation (40 psi) of diol-d8 **5** over palladium on carbon (5 wt. %) gave the amine CP-506M-(OH)_2_-8D.

**Supplementary Scheme 3.** Synthetic route to CP-506H-(OH)_2_-8D and CP-506M-(OH)_2_-8D

CP-506H-Cl_2_-8D and CP-506M-Cl_2_-8D, the octa-deuterated standards of the parent compounds, were synthesised according to the route shown in Supplementary Scheme 4. Briefly, reaction of diol-d8 **5** with methanesulfonyl chloride and triethylamine in dichloromethane at 0 ^o^C gave the bis-mesylate-d8 **6**. Lithium chloride mediated displacement of the mesylate groups of bis-mesylate-d8 **6** in acetone at room temperature gave the bis-chloro mustard-d8 **7**. Hydrogenation (40 psi) of mustard-d8 **7** over Raney®-Nickel (activated catalyst, 50% slurry in water) gave the hydroxylamine CP-506H-Cl_2_-8D. Alternately, hydrogenation (40 psi) of mustard-d8 **7** over palladium on carbon (5 wt. %) gave the amine CP-506M-Cl_2_-8D.

**Supplementary Scheme 4.** Synthetic route to CP-506H-Cl_2_-8D and CP-506M-Cl_2_-8D

**Chemistry experimental**

^1^H NMR spectra were measured on a Bruker Avance‑400 spectrometer and are referenced to Me_4_Si. High resolution mass spectra were recorded on a Varian VG-70SE spectrometer at nominal 5000 resolution. Mass spectrometry was performed on a ThermoFinnigan MSQ single quadrupole mass spectrometer. Mass detection was performed with an APCI source, using simultaneous positive and negative ion acquisition. Unless otherwise indicated, compounds were purified by flash column chromatography on Silica gel 60 support (Scharlau, 230-400 mesh ASTM), using the indicated eluants.

*(5-(Bis(2-hydroxyethyl)amino)-4-(methylsulfonyl)-2-nitrophenyl)(4-ethylpiperazin-1-yl)methanone (****2****)*

(4-Ethylpiperazin-1-yl)(5-fluoro-4-(methylsulfonyl)-2-nitrophenyl)methanone **1** (2.28 g, 6.34 mmol) was dissolved in DMSO (10 mL) and treated with diethanolamine (1.0 g, 9.51 mmol). The reaction mixture was stirred at room temperature overnight then poured into ice/water and extracted with EtOAc (3x). The combined organic phase was washed with brine, dried over anhydrous Na_2_SO_4_ and concentrated to dryness under reduced pressure. The residue was purified by flash column chromatography on silica gel eluting with DCM/MeOH (17:3) containing 2% conc. NH_3_ to provide (5-(bis(2-hydroxyethyl)amino)-4-(methylsulfonyl)-2-nitrophenyl)(4-ethylpiperazin-1-yl)methanone **2** (1.57 g, 56%) as a yellow gum. ^1^H NMR [(CD_3_)_2_SO] δ 8.62 (s, 1H), 7.45 (s, 1H), 4.60 (t, *J* = 4.9 Hz, 2H), 3.75-3.65 (m, 1H), 3.57-3.47 (m, 9H), 3.46 (s, 3H), 3.22-3.10 (m, 3H), 2.42-2.33 (m, 4H), 2.23-2.18 (m, 1H), 1.00 (t, *J* = 7.15 Hz, 3H). LRMS (APCI^+^) *m/z*: calcd for C_18_H_28_N_4_O_7_S, 444; found [M+H]^+^, 445.

*(5-(Bis(2-hydroxyethyl)amino)-2-(hydroxyamino)-4-(methylsulfonyl)phenyl)(4-ethylpiperazin-1-yl)methanone (****CP-506H-(OH)_2_****)*

(5-(Bis(2-hydroxyethyl)amino)-4-(methylsulfonyl)-2-nitrophenyl)(4-ethylpiperazin-1-yl)methanone **2** (150 mg, 0.34 mmol) was dissolved in EtOAc/THF (1:1, 14 mL) and treated with Raney®-Nickel (49 mg, 0.84 mmol). The reaction mixture was degassed and stirred under an atmosphere of H_2_ (40 psi) overnight then passed through a short pad of celite. The solvents were removed under reduced pressure at room temperature. The residue was purified by flash column chromatography on neutral alumina eluting with DCM/MeOH (9:1) to provide the desired product, CP-506H-(OH)_2_ (82 mg, 56%) as an unstable yellow gum. ^1^H NMR (CDCl_3_) δ 7.95 (s, 1H), 7.48 (br s, 1H), 7.24 (s, 1H), 3.82-3.75 (m, 2H), 3.70-3.68 (m, 5H), 3.41 (s, 3H), 3.40-3.32 (m, 3H), 3.14 (t, *J* = 5.11 Hz, 4H), 2.54-2.41 (m, 6H), 1.12 (t, *J* = 7.22 Hz, 3H). LRMS (APCI^+^) *m/z*: calcd for C_18_H_30_N_4_O_6_S, 430; found [M+H]^+^, 431.

*(2-Amino-5-(bis(2-hydroxyethyl)amino)-4-(methylsulfonyl)phenyl)(4-ethylpiperazin-1-yl)methanone (****CP-506M-(OH)_2_****)*

(5-(Bis(2-hydroxyethyl)amino)-4-(methylsulfonyl)-2-nitrophenyl)(4-ethylpiperazin-1-yl)methanone **2** (150 mg, 0.34 mmol) was dissolved in EtOAc/THF (1:1, 14 mL) and treated with Pt/C (5 wt. %, 4 mol %). The reaction mixture was degassed and stirred under an atmosphere of H_2_ (40 psi) overnight then passed through a short pad of celite. The solvents were removed under reduced pressure at room temperature. The residue was purified by flash column chromatography on neutral alumina eluting with DCM/MeOH (9:1) to provide the desired product, CP-506M-(OH)_2_ (130 mg, 93%) as a yellow gum. ^1^H NMR (CDCl_3_) δ 7.40 (s, 1H), 7.22 (s, 1H), 4.42 (s, 2H), 3.68 (br s, 6H), 3.49 (br s, 4H), 3.40 (s, 3H), 3.12-3.10 (m, 4H), 2.48-2.43 (m, 6H), 1.10 (t, *J* = 7.22 Hz, 3H). LRMS (APCI^+^) *m/z*: calcd for C_18_H_30_N_4_O_5_S, 414; found [M+H]^+^, 415.

*(5-(Bis(2-((methylsulfonyl)oxy)ethyl)amino)-4-(methylsulfonyl)-2-nitrophenyl)(4-ethyl-1-piperazine-1-yl)methanone (****3****)*

(5-(Bis(2-hydroxyethyl)amino)-4-(methylsulfonyl)-2-nitrophenyl)(4-ethylpiperazin-1-yl)methanone **2** (800 mg, 1.80 mmol) was dissolved in DCM (80 mL), cooled to 0 °C and treated with Et_3_N (1.1 mL, 7.89 mmol) then MsCl (505 μL, 6.52 mmol). The reaction mixture was stirred at 0 °C for 1 h then neutralized by addition of a saturated solution of NaHCO_3_. The organic phase was washed with water, dried over anhydrous Na_2_SO_4_ and concentrated to dryness under reduced pressure. The crude (5-(bis(2-((methylsulfonyl)oxy)ethyl)amino)-4-(methylsulfonyl)-2-nitrophenyl)(4-ethyl-1-piperazine-1-yl)methanone (**3**) was used in the next step without further purification.

*(5-(Bis(2-chloroethyl)amino)-4-(methylsulfonyl)-2-nitrophenyl)(4-ethylpiperazin-1-yl)methanone (****4****)*

(5-(Bis(2-((methylsulfonyl)oxy)ethyl)amino)-4-(methylsulfonyl)-2-nitrophenyl)(4-ethyl-1-piperazine-1-yl)methanone **3** (1.0 g, 1.66 mmol) was dissolved in acetone (120 mL) and treated with LiCl (1.41 g, 33.2 mmol). The reaction mixture was stirred at room temperature overnight and the solvent was removed under reduced pressure. The residue was dissolved in EtOAc and washed with water (2x). The organic phase was dried over anhydrous Na_2_SO_4_ and concentrated to dryness under reduced pressure. The crude product was purified by flash column chromatography on silica gel eluting with DCM/MeOH (19:1) to provide (5-(bis(2-chloroethyl)amino)-4-(methylsulfonyl)-2-nitrophenyl)(4-ethylpiperazin-1-yl)methanone **4** (526 mg, 66%) as a yellow gum. ^1^H NMR [(CD_3_)_2_SO] δ 8.65 (s, 1H), 7.65 (s, 1H), 3.78 (m, 8H), 3.69-3.52 (m, 2H), 3.47 (s, 3H), 3.18 (m, 2H), 2.55 (m, 1H), 2.42 (m, 1H), 2.39-2.33 (m, 3H), 2.21 (m, 1H), 1.00 (t, *J* = 7.15 Hz, 3H). LRMS (APCI^+^) *m/z*: calcd for C_18_H_26_Cl_2_N_4_O_5_S, 480; found [M+H]^+^, 481.

*(5-(Bis(2-chloroethyl)amino)-2-(hydroxyamino)-4-(methylsulfonyl)phenyl)(4-ethylpiperazin-1-yl)methanone (****CP-506H-Cl_2_****)*

Hydrogenation of a solution of compound **4** (150 mg, 0.31 mmol) in EtOAc/THF using H_2_ gas (40 psi) over Raney®-Nickel according to the previously described method provided CP-506H-Cl_2_ (96 mg, 66%) as an unstable yellow gum. ^1^H NMR (CDCl_3_) δ 8.03 (s, 1H), 7.52 (br s, 1H), 7.19 (s, 1H), 6.43 (br s, 1H), 3.80 (m, 2H), 3.60 (m, 8H), 3.38 (m, 2H), 3.36 (s, 3H), 2.54 (m, 2H), 2.48 (q, *J* = 7.22 Hz, 2H), 2.42 (m, 2H), 1.12 (t, *J* = 7.22 Hz, 3H). LRMS (APCI^-^) *m/z*: calcd for C_18_H_28_Cl_2_N_4_O_4_S, 466; found [M-H]^-^, 465.

*(2-Amino-5-(bis(2-chloroethyl)amino)-4-(methylsulfonyl)phenyl)(4-ethylpiperazin-1-yl)methanone (****CP-506M-Cl_2_****)*

Hydrogenation of a solution of compound **4** (100 mg, 0.21 mmol) in EtOAC/THF using H_2_ gas (40 psi) over Pd/C (5 wt. %) according to the previously described method provided CP-506M-Cl_2_ (53 mg, 57%) as an unstable yellow gum. ^1^H NMR (CDCl_3_) δ 7.46 (s, 1H), 7.16 (s, 1H), 5.01 (s, 2H), 3.61-3.59 (m, 6H), 3.55-3.52 (m, 6H), 3.31 (s, 3H), 2.49-2.43 (m, 6H), 1.11 (t, *J* = 7.22 Hz, 3H). LRMS (APCI^+^) *m/z*: calcd for C_18_H_28_Cl_2_N_4_O_3_S, 450; found [M+H]^+^, 451.

*(5-(bis(2-hydroxyethyl-1, 1, 2, 2-d_4_)amino)-4-(methylsulfonyl)-2-nitrophenyl)(4-ethylpiperazin-1-yl)methanone (****5****)*

Reaction of (4-ethylpiperazin-1-yl)(5-fluoro-4-(methylsulfonyl)-2-nitrophenyl)methanone **1** (2.75 g, 6.95 mmol) with diethanolamine-d8 in DMSO according to the previously described method provided (5-(bis(2-hydroxyethyl-1, 1, 2, 2-*d_4_*)amino)-4-(methylsulfonyl)-2-nitrophenyl)(4-ethylpiperazin-1-yl)methanone **5** (1.66 g, 48%) as a yellow gum. ^1^H NMR [(CD_3_)_2_SO] δ 8.62 (s, 1H), 7.44 (s, 1H), 4.66 (s, 2H), 3.69 (m, 1H), 3.55 (m, 1H), 3.45 (s, 3H), 3.19-3.15 (m, 3H), 2.41 (m, 1H), 2.39-2.33 (m, 3H), 2.22 (m, 1H), 0.98 (t, *J* = 7.16 Hz, 3H). LRMS (APCI^+^) *m/z*: calcd for C_18_H_20_D_8_N_4_O_7_S, 452; found [M+H]^+^, 453.

*(5-(Bis(2-hydroxyethyl-1,1,2,2-d4)amino)-2-(hydroxyamino)-4-(methylsulfonyl)phenyl)(4-ethylpiperazin-1-yl)methanone (****CP-506H-(OH)_2_-8D****)*

Hydrogenation of a solution of compound **5** (139 mg, 0.31 mmol) in EtOAC/THF using H_2_ gas over Raney®-Nickel according to the previously described method provided CP-506H-(OH)_2_-8D (74 mg, 55%) as an unstable yellow gum. ^1^H NMR (CDCl_3_) δ 7.97 (s, 1H), 7.54 (br s, 1H), 7.24 (s, 1H), 5.84 (br s, 1H), 3.81-3.77 (m, 2H), 3.42 (s, 3H), 3.38 (m, 4H), 2.53 (m, 2H), 2.46 (q, *J* = 7.23 Hz, 2H), 2.40 (m, 2H), 1.11 (t, *J* = 7.22 Hz, 3H). LRMS (APCI^+^) *m/z*: calcd for C_18_H_22_D_8_N_4_O_6_S, 438; found [M+H]^+^, 439.

*(2-Amino-5-(bis(2-hydroxyethyl-1,1,2,2-d4)amino)-4-(methylsulfonyl)phenyl)(4-ethylpiperazin-1-yl)methanone (****CP-506M-(OH)_2_-8D****)*

Hydrogenation of a solution of compound **5** (108 mg, 0.24 mmol) in EtOAC/THF using H_2_ gas over Pd/C (5 wt. %) according to the previously described method provided CP-506M-(OH)_2_-8D (88 mg, 88%) as a yellow gum. ^1^H NMR (CDCl_3_) δ 7.40 (s, 1H), 7.21 (s, 1H), 5.01 (s, 2H), 3.70 (m, 2H), 3.40 (s, 3H), 3.30 (m, 2H), 3.08 (m, 2H), 2.54-2.43 (m, 6H), 1.11 (t, *J* = 7.22 Hz, 3H). LRMS (APCI^+^) *m/z*: calcd for C_18_H_22_D_8_N_4_O_5_S, 422; found [M+H]^+^, 423.

*(5-(Bis(2-((methylsulfonyl)oxy)ethyl-1, 1, 2, 2-d_4_)amino)-4-(methylsulfonyl)-2-nitrophenyl)(4-ethyl-1-piperazine-1-yl)methanone (****6****)*

(5-(Bis(2-hydroxyethyl-1, 1, 2, 2-*d_4_*)amino)-4-(methylsulfonyl)-2-nitrophenyl)(4-ethylpiperazin-1-yl)methanone **5** (805 mg, 1.78 mmol) in DCM was cooled to 0 °C before being reacted with MsCl and Et_3_N according to the previously described method to provide (5-(bis(2-((methylsulfonyl)oxy)ethyl-1, 1, 2, 2-*d_4_*)amino)-4-(methylsulfonyl)-2-nitrophenyl)(4-ethyl-1-piperazine-1-yl)methanone **6** as a yellow gum. The crude product **6** was used in the next step without further purification.

*(5-(Bis(2-chloroethyl-1, 1, 2, 2-d_4_)amino)-4-(methylsulfonyl)-2-nitrophenyl)(4-ethylpiperazin-1-yl)methanone (****7****)*

A solution of compound **6** (1.08 g, 1.78 mmol) in acetone was reacted with LiCl according to the previously described method to give (5-(bis(2-chloroethyl-1, 1, 2, 2-*d_4_*)amino)-4-(methylsulfonyl)-2-nitrophenyl)(4-ethylpiperazin-1-yl)methanone **7** (630 mg, 78%) as a yellow gum. ^1^H NMR [(CD_3_)_2_SO] δ 8.65 (s, 1H), 7.67 (s, 1H), 3.69 (m, 1H), 3.58 (m, 1H), 3.46 (s, 3H), 3.18 (m, 2H), 2.43 (m, 1H), 2.39-2.34 (m, 4H), 2.22 (m, 1H), 1.00 (t, *J* = 7.16 Hz, 3H). LRMS (APCI^+^) *m/z*: calcd for C_18_H_18_D_8_Cl_2_N_4_O_5_S, 488; found [M+H]^+^, 489.

*(5-(Bis(2-chloroethyl-1,1,2,2-d4)amino)-2-(hydroxyamino)-4-(methylsulfonyl)phenyl)(4-ethylpiperazin-1-yl)methanone (****CP-506H-Cl_2_-8D****)*

Hydrogenation of a solution of compound **7** (130 mg, 0.27 mmol) in EtOAc/THF using H_2_ gas over Raney®-Nickel according to the previously described method provided CP-506H-Cl_2_-8D (63 mg, 50%) as an unstable yellow gum. ^1^H NMR (CDCl_3_) δ 7.82 (s, 1H), 7.54 (br s, 1H), 7.28 (s, 1H), 6.76 (br s, 1H), 3.30 (m, 2H), 3.66 (m, 2H), 3.33 (s, 3H), 2.46 (m, 2H), 2.39 (q, *J* = 7.21 Hz, 2H), 2.35 (m, 2H), 1.03 (t, *J* = 7.20 Hz, 3H). LRMS (APCI^-^) *m/z*: calcd for C_18_H_20_D_8_Cl_2_N_4_O_4_S, 474; found [M-H]^-^, 473.

*(2-Amino-5-(bis(2-chloroethyl-1,1,2,2-d4)amino)-4-(methylsulfonyl)phenyl)(4-ethylpiperazin-1-yl)methanone (****CP-506M-Cl_2_-8D****)*

Hydrogenation of a solution of compound **7** (132 mg, 0.27 mmol) in EtOAc/THF using H_2_ gas over Pt/C (5 wt. %) according to the previously described method provided CP-506M-Cl_2_-8D (94 mg, 76%) as an unstable yellow gum. ^1^H NMR (CDCl_3_) δ 7.46 (s, 1H), 7.15 (s, 1H), 4.42 (s, 2H), 3.60 (m, 4H), 3.35 (s, 3H), 2.49-2.43 (m, 6H), 1.10 (t, *J* = 7.22 Hz, 3H). LRMS (APCI^+^) *m/z*: calcd for C_18_H_20_D_8_Cl_2_N_4_O_3_S, 458; found [M+H]^+^, 459.

1. **SUPPLEMENTARY TABLES**

**Supplementary Table S1.** Antibodies and blocking agents used for immunoblotting.

| Protein | Antibody Type | Source | Blocking agent | Dilution |
| --- | --- | --- | --- | --- |
| POR | Mouse monoclonal | Santa Cruz Biotechnology, Dallas, TX, USA | 5% BSA in TBS-T | 1: 1000 |
| β-actin | Mouse monoclonal | Sigma-Aldrich, St. Louis, MO, USA | 5% BSA in TBS-T | 1: 5000 |
| - | Goat anti-mouse IgG-HRP | Santa Cruz Biotechnology, Dallas, TX, USA | 5% BSA in TBS-T | 1: 5000 |

**Supplementary Table S2.** MRM transition monitored for each test compound.

| Compound | MRM transition (s) | Ionization mode | Collison energy (eV) | Dwell time (ms) | Fragmentor voltage  (V) | Retention time (min) |
| --- | --- | --- | --- | --- | --- | --- |
| CP-506 | 586.9>491.0 | ESI+ | 14 | 100 | 120 | 3.8 |
| CP-506H | 553.2>457.2 | ESI+ | 12 | 100 | 125 | 2.7 |
| CP-506M | 557.2>461.2 | ESI+ | 12 | 100 | 125 | 3.0 |
| CP-506H-Cl_2_ | 466.9>353 | ESI+ | 18 | 100 | 140 | 3.9 |
| CP-506H-(OH)_2_ | 431>220.9 | ESI+ | 21 | 100 | 130 | 0.9 |
| CP-506M-Cl_2_ | 451>220.9 | ESI+ | 30 | 100 | 115 | 4.1 |
| CP-506M-(OH)_2_ | 415>220.9 | ESI+ | 25 | 100 | 140 | 0.9 |
| CP-506 D8 | 594.9>499.0 | ESI+ | 14 | 100 | 120 | 3.8 |
| CP-506H D8 | 561.2>465.2 | ESI+ | 12 | 100 | 125 | 2.7 |
| CP-506M D8 | 565.2>469.2 | ESI+ | 12 | 100 | 125 | 3.0 |
| CP-506H-Cl_2_ D8 | 474.9>361 | ESI+ | 18 | 100 | 140 | 3.9 |
| CP-506H-(OH)_2_ D8 | 439>228.9 | ESI+ | 21 | 100 | 130 | 0.9 |
| CP-506M-Cl_2_ D8 | 459>228.9 | ESI+ | 30 | 100 | 115 | 4.1 |
| CP-506M-(OH)_2_ D8 | 423>228.9 | ESI+ | 25 | 100 | 140 | 0.9 |

**Supplementary Table S3.** Parameter estimates for the Cellular PK model. Values were estimated by fitting the accrued cellular uptake data with the first order kinetic model depicted in Figure 4 using the monolayer ABM. Terms to describe the cellular uptake (k_in(4)_), cellular efflux (k_out(4)_) and extracellular instability (T_1/2(4)_) of CP-506M-Cl_2_ were fixed to the fitted values for the downstream metabolites of PR-104 (termed ‘metabolite 2’), as reported previously (2,3).

| Parameter | Estimate | Units | Description |
| --- | --- | --- | --- |
| k_in(1)_ | 3.7 | min^-1^ | Rate of transfer for CP-506 from the extracellular to the intracellular compartment |
| k_out(1)_ | 0.06 | min^-1^ | Rate of transfer for CP-506 from the intracellular to the extracellular compartment |
| k_met(1)_ | 0.12 | min^-1^ | Rate of metabolism for CP-506 to CP-506H |
| k_in(2)_ | 0.9 | min^-1^ | Rate of transfer for CP-506H from the extracellular to the intracellular compartment |
| k_out(2)_ | 0.25 | min^-1^ | Rate of transfer for CP-506H from the intracellular to the extracellular compartment |
| k_met(2)_ | 0.09 | min^-1^ | Rate of metabolism for CP-506H to CP-506M |
| k_in(3)_ | 0.9 | min^-1^ | Rate of transfer for CP-506M from the extracellular to the intracellular compartment |
| k_out(3)_ | 0.4 | min^-1^ | Rate of transfer for CP-506M from the intracellular to the extracellular compartment |
| k_met(3)_ | 0.1 | min^-1^ | Rate of metabolism for CP-506M to CP-506M-Cl_2_ |
| k_in(4)_ | 0.9 | min^-1^ | Rate of transfer for CP-506M-Cl_2_ from the extracellular to the intracellular compartment |
| k_out(4)_ | 0.3 | min^-1^ | Rate of transfer for CP-506M-Cl_2_ from the intracellular to the extracellular compartment |
| T_1/2 (1)_ | 20 | hours | Time required to reduce the concentration of CP-506 to half its initial value |
| T_1/2 (2)_ | 0.21 | hours | Time required to reduce the concentration of CP-506H to half its initial value |
| T_1/2 (3)_ | 0.11 | hours | Time required to reduce the concentration of CP-506M to half its initial value |
| T_1/2 (4)_ | 0.11 | hours | Time required to reduce the concentration of CP-506M-Cl_2_ to half its initial value |

**Supplementary Table S4.** Diffusion parameters for CP-506 through POR-R MCLs under supraoxic and anoxic conditions. Values are the mean ± SEM with the number of repeats in parentheses.

| Parameter | Units | Gas phase | | Description |
| --- | --- | --- | --- | --- |
|  |  | **Supraoxia** | **Anoxia** |  |
| C_0_ ^a^ | µM | 18.3 ± 0.4 | 17.9 ± 4.7 | Initial concentration |
| D_sup_ | x 10^-6^ cm^2^ s^-1^ | 1.32 ± 0.19 (2) ^b^ | 0.717 ± 0.01 (3) | Diffusion coefficient across bare support membranes |
| L_MCL_ | µm | 93.98 ± 9.8 (3) | 109.46 ± 9.4 (3) | Estimated MCL thickness |
| D_MCL_ | x 10^-6^ cm^2^ s^-1^ | 1.93 ± 0.01 (6) | 1.93 (fixed) | Diffusion coefficient across POR-R MCLs |
| φ_i_ ^c^ | - | - | 0.3 | Metabolic scaling factor |
| ^a^ Initial concentration (C_o_) calculated as the average mass balance (supraoxia) or by extrapolation by linear regression from the concentration-time profile of CP-506 in the donor compartment (anoxia).  ^b^ A diffusion coefficient was unable to be fitted for one replicate due to the slower stirring rate. Individual model fits for the accrued data have been provided in Supplementary Figure S3.  ^c^ Metabolic scaling factor for refining parameter estimates between monolayer cultures and tissue-like density. | | | | |

1. **SUPPLEMENTARY FIGURES**


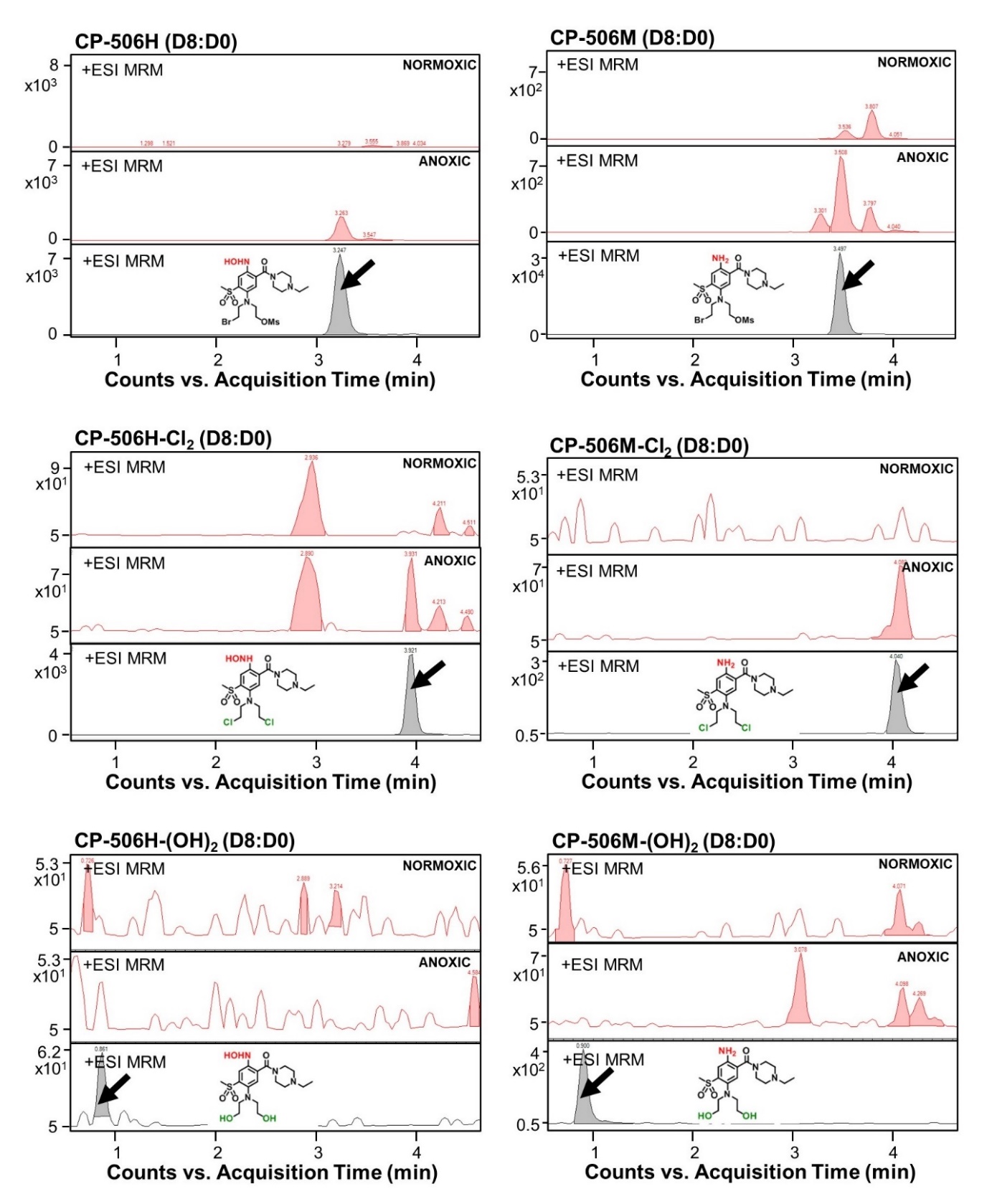


**Supplementary Figure S1.** LC-MS/MS traces for CP-506 metabolites. Spectra of CP-506M, CP-506M-Cl_2_, CP-506M-(OH)_2_, CP-506H, CP-506H-Cl_2_ and CP-506H-(OH)_2_ following exposure to 100 µM CP-506 for 1 h under normoxic (21% O_2_) and anoxic conditions (< 1 ppm O_2_). Samples (red spectra) were compared to the corresponding standard (black spectra) and the expected mass is annotated with the black arrow.


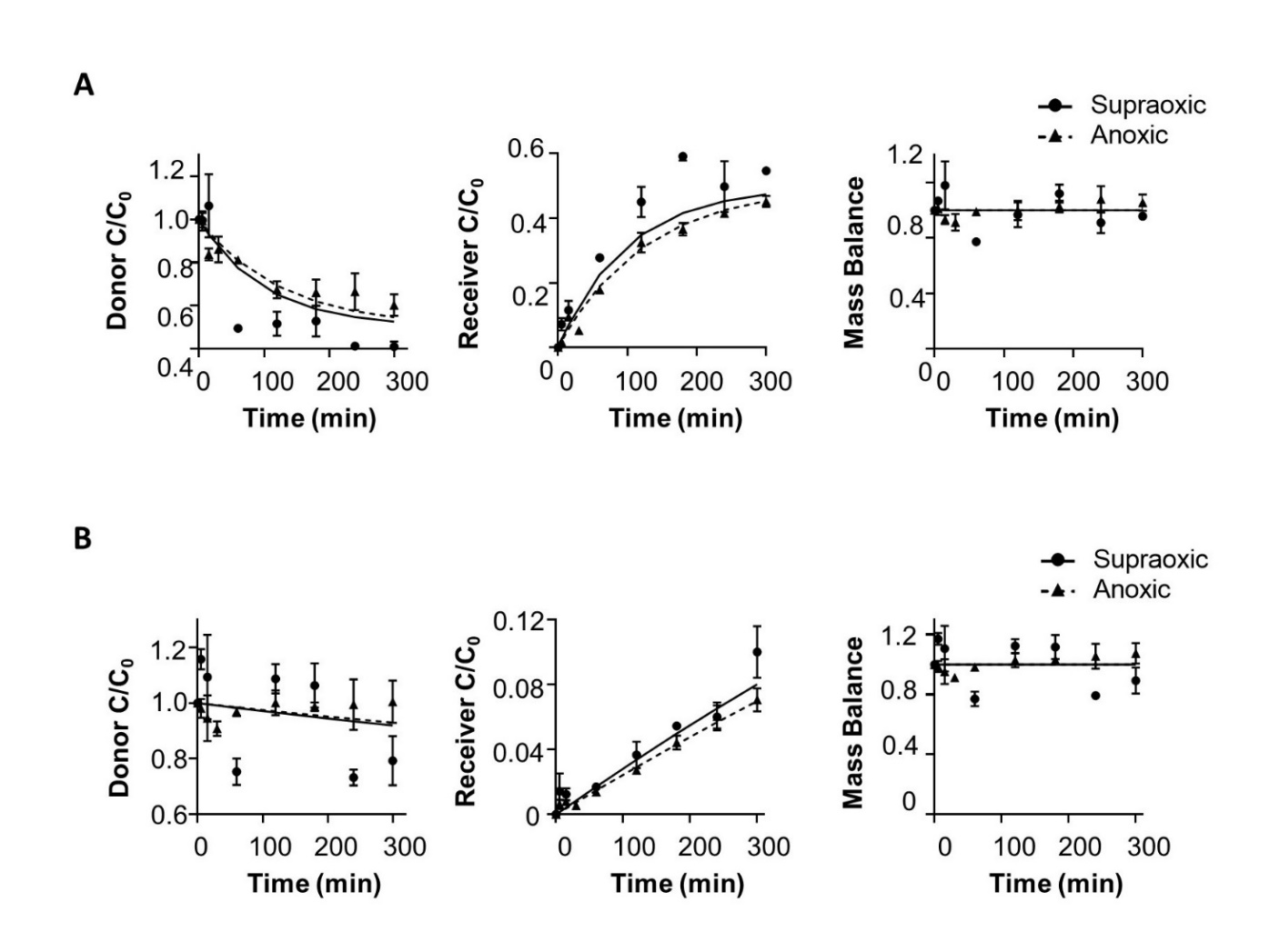


**Supplementary Figure S2.** Extravascular transport of [^14^C]-urea across support membranes with or without an MCL culture. Flux was initiated by the addition of CP-506 (C_o_ = 17.4 ± 1.3 µM) and internal standard [^14^C]-urea into the donor compartment of diffusion chambers maintained under supraoxic (95% O_2_, 5% CO_2_) and anoxic (5% CO_2_, bal N_2_) conditions. The concentration-time profile of [^14^C]-urea in the donor and receiver compartments of diffusion chambers was determined by mass spectrometry. Mass balance was calculated as the sum of the donor and receiver compartments. Lines are the concentrations predicted from the developed transport model. Values are mean ± SEM of three independent diffusion chambers. **(A)** Concentration-time profile of [^14^C]-urea across bare support membranes (without cells) maintained under supraoxic and anoxic conditions. **(B)** Concentration-time profile of [^14^C]-urea across POR-R MCLs maintained under supraoxic and anoxic conditions.


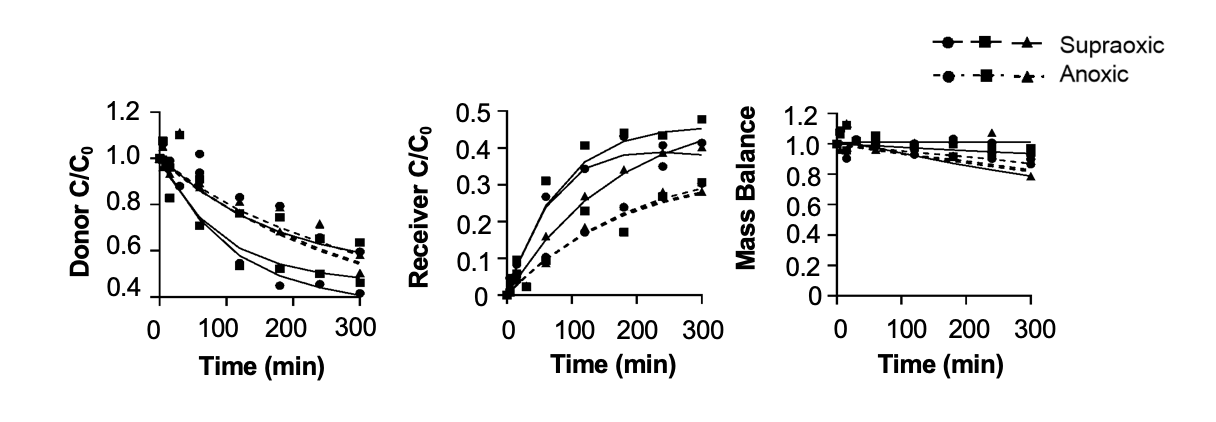


**Supplementary Figure S3.** Individual model fits for the diffusion of CP-506 across the bare support membrane under supraoxic and anoxic conditions. Flux was initiated by the addition of CP-506 (C_o_ = 17.4 ± 1.3 µM) and internal standard [^14^C]-urea into the donor compartment of diffusion chambers maintained under supraoxic (95% O_2_, 5% CO_2_) and anoxic (5% CO_2_, bal N_2_) conditions. The concentration-time profile of CP-506 in the donor and receiver compartments of diffusion chambers was determined by mass spectrometry. Mass balance was calculated as the sum of the donor and receiver compartments. Symbols show the concentration-time profile of CP-506 in the donor and receiver compartment of three independent diffusion chambers. Lines are the concentrations predicted from the developed (pro)drug transport model.

1. **REFERENCES**

1. Van Der Wiel AMA, Jackson-Patel V, Niemans R, Yaromina A, Liu E, Marcus D, et al. Selectively Targeting Tumor Hypoxia with the Hypoxia-Activated Prodrug CP-506. Mol Cancer Ther. 2021. Available from: https://doi.org/10.1158/1535-7163.MCT-21-0406

2. Foehrenbacher A, Patel K, Abbattista MR, Guise CP, Secomb TW, Wilson WR, et al. The role of bystander effects in the anti-tumour activity of the hypoxia-activated prodrug PR-104A. Front Oncol. 2013;3:1–18. Available from: https://doi.org/10.3389/fonc.2013.00263

3. Hong CR, Bogle G, Wang J, Patel K, Pruijn FB, Wilson WR, et al. Bystander effects of hypoxia-activated prodrugs: Agent-based modeling using three dimensional cell cultures. Front Pharmacol. 2018;9:1–16. Available from: https://doi.org/10.3389/fphar.2018.01013
